# Association of short-chain fatty acid–producing gut microbiota and dietary habits with maternal depression in a subclinical population

**DOI:** 10.1093/pnasnexus/pgaf169

**Published:** 2025-09-02

**Authors:** Michiko Matsunaga, Mariko Takeuchi, Satoshi Watanabe, Aya K Takeda, Keisuke Hagihara, Masako Myowa

**Affiliations:** Department of Advanced Hybrid Medicine, Graduate School of Medicine, Osaka University, Osaka 565-0871, Japan; Graduate School of Education, Kyoto University, Kyoto 606-8501, Japan; Japan Society for the Promotion of Science, Tokyo 102-0083, Japan; Department of Advanced Hybrid Medicine, Graduate School of Medicine, Osaka University, Osaka 565-0871, Japan; Cykinso, Inc., Tokyo 151-0053, Japan; Cykinso, Inc., Tokyo 151-0053, Japan; Department of Advanced Hybrid Medicine, Graduate School of Medicine, Osaka University, Osaka 565-0871, Japan; Graduate School of Education, Kyoto University, Kyoto 606-8501, Japan

**Keywords:** depression, mothers, intestinal microbiome, diet, fermented food

## Abstract

The prevalence of postpartum mental illness is steadily increasing, a tendency that was exacerbated by the COVID-19 pandemic. Recent studies show that maternal depression is no longer confined to the perinatal period, and this necessitates long-term assessment and support for maternal mental health. It is critical to identify the factors that are related to depression among mothers, and this requires the development of integrated mental and physical health care encompassing both psychological aspects and intestinal microbiota, physical conditions, and dietary habits. Studies conducted in western countries have examined the association between gut microbiota and depressive disorders. However, little is known concerning postpartum mothers in healthy populations. In addition, even in healthy populations, some mothers will have severe depression. This is because mothers in Japan are typically hesitant to disclose psychiatric symptoms and tend not to consult specialists. Therefore, we conducted a cross-sectional study to investigate the association of intestinal microbiota, physical condition, and dietary habits with depressed mood in healthy mothers in Japan. We found that microbiome diversity (Shannon *α*) and relative abundance of butyrate-producing bacteria (e.g. *Lachnospira*, *Faecalibacterium*, and *Subdoligranulum*), obtained using 16S rRNA gene-sequencing analysis, were associated with high levels of depressive mood. Mothers who have this attribute showed poorer sleep quality and worse physical condition than mothers with low levels of depressive mood. The evaluation of dietary habits suggested that dietary patterns high in soy products, fermented food, seaweed, and mushrooms, as well as vegetables, are beneficial for depression and intestinal microbiota (e.g. *Lachnospira*, *Agathobacter*, and *Subdoligranulum*).

Significance Statement Studies have examined the association between gut microbiota and depressive mood in a subclinical population. Significant numbers of mothers have a severely depressive mood even in a healthy population. However, little is known concerning postpartum mothers in healthy populations. We show that microbiota diversity (Shannon *α*) and relative abundance of butyrate-producing bacteria (e.g. *Lachnospira*, *Faecalibacterium*, and *Subdoligranulum*) obtained by 16S rRNA gene-sequencing analysis were associated with high levels of depressive mood. The evaluation of dietary habits suggests that dietary patterns high in soy products, fermented food, seaweed, and mushrooms, as well as vegetables, are beneficial for mental and physical health, including depression and healthy intestinal microbiota (e.g. *Lachnospira, Agathobacter*, and *Subdoligranulum*).

## Introduction

In developed countries, 10–15% of women suffer from postpartum depression (1, 2), and this proportion increased following the COVID-19 pandemic (3–6). The latest data show that currently, around 25–30% of mothers have postpartum depression (7). In recent years, moreover, postpartum depression is no longer limited in time to the perinatal period but can persist for long periods (8, 9). A recent study found that women are more likely to experience depression in the first 4 years postpartum than during the first year (10). This indicates the need to assess and support the mother's mental health, not only during the brief perinatal period but also over the long term, at least partially because mothers' prolonged depression can affect children's mental health and cognitive development (11, 12). It is crucial to investigate the factors contributing to reducing or preventing mothers' depression and reflecting those factors in primary health care.

Depression is not only manifested in psychological symptoms such as decreased motivation and depressed mood but also in sleep disturbances (e.g. insomnia and hypersomnia), anorexia, and worsening physical condition (e.g. malaise, headache, heart palpitations, dizziness, and tinnitus) (13–16). In the primary care phase, 69% of depression patients reported only physical symptoms (13, 17), likely because mothers hesitated to disclose psychological symptoms (18). In other words, it is not easy to screen for mothers' depressive symptoms (18). For this reason, we need to comprehensively examine the physical and physiological conditions that are associated with signs of depressive symptoms in healthy mothers (i.e. those who are not diagnosed with or under treatment for mental and/or physical illness) to identify the relevant factors that can help prevent or reduce depressive mood and support intervention before depressive disorders worsen.

In this context, the intestinal microbiota has recently received attention as a neurophysiological factor related to depression. Recent studies have shown that it is not only associated with physical diseases (e.g. diabetes and cancer) but also with psychological disorders (e.g. depression and anxiety) (19, 20). For example, a recent systematic review found that patients with depression and anxiety disorders have more inflammatory gut bacteria and relatively fewer short-chain, fatty acid–producing gut bacteria (21–23). The intestinal microbiota affects brain functions through the autonomic nervous system, neuropeptides, hormones, and the immune system (24). Investigating the intestinal microbiota can contribute to developing a more detailed understanding of the relationship between physical symptoms, daily dietary habits, and psychological depression. Furthermore, this investigation could contribute to the development of methods of preventing adverse mental health and methods of intervention using the intestinal microbiota (e.g. improving dietary habits and administering probiotics) (25).

The association between intestinal microbiota and depression has generally received the most attention in clinical samples of western countries (in particular, in those who have been diagnosed with depressive disorders and are under treatment). Still, studies of mothers remain limited (22, 23). In addition, no large-scale studies in healthy populations have aimed at the early detection of undiagnosed, untreated depression and the prevention of severe psychiatric disorders (26, 27). In the context of our study, it is important to note that the intestinal microbiota of the Japanese population differs from that of western populations and even from those of other Asian populations (e.g. populations in China) (28). It is particularly important for our study to consider Japanese food culture and dietary habits, as these can influence the state of intestinal microbiota (29, 30). The Japanese diet positively affects cognitive function and physical health (30, 31). Recent studies in nutritional epidemiology have shown that eating habits that are based on the consumption of vegetables, fruits, and fish are associated with reduced depression. In contrast, eating a diet that is high in high-fat dairy products and processed foods is associated with depression (32–35). Dietary patterns vary considerably by gender, socioeconomic status, and culture (33, 36); however, high-quality dietary patterns in the Japanese population may contain different food components than those found in western populations (29, 30, 35, 37). For example, such patterns in Japan include vegetables, fruits, fish/shellfish, mushrooms, seaweed, soy products, and potatoes, all of which are associated with reduced depressive symptoms (35, 37). These dietary patterns may contribute to the composition of the intestinal microbiota and declines in depressive mood. Thus, we investigated the relationship between the intestinal microbiota and the physical and mental health of mothers, taking into account the Japanese dietary culture and eating habits.

This study is cross-sectional in nature and analyzes stool samples of 344 mothers caring for infants and toddlers aged 0–4 years attending preschool or kindergarten in Japan; we also examined factors of intestinal microbiota, physical condition (i.e. physical symptoms, sleep duration, and sleep quality), and dietary habits associated with depressive mood. With respect to intestinal microbiota, we analyzed their diversity (i.e. Shannon *α* and beta diversity using unweighted and weighted UniFrac distances) and the prevalent composition of the microbiota with 16S rRNA gene-sequencing analysis. We hypothesized that intestinal microbiota is associated with depressive mood as assessed using the Beck Depression Inventory-II (BDI-II) (38, 39) and that mothers with high levels of depressive mood would have lower microbiota diversity and fewer short-chain fatty acid–producing enterobacteria. We also compared groups having high and low scores for depression using the BDI-II cutoff score and compared mothers' physical condition based on three measurements (i.e. sleep duration, sleep quality, and indices of the multidimensional physical scale [MDPS] (40)) and dietary habits. We further hypothesized that mothers with a high score of depression would have poorer physical conditions than mothers with low scores for depression and be more unbalanced in their meals' frequency and variety. Finally, we examined the relationship between dietary patterns and their effects on the depressive mood, physical condition, and intestinal microbiota of the mothers. We identified dietary patterns using factor analysis and compared the high- and low-score groups using the median as the cutoff. We explored the effects of dietary patterns on depressive mood, physical condition, and intestinal microbiota.

## Results

### Relationships between depressed mood, dietary and lifestyle habits, and intestinal microbiota among mothers raising children aged 0–4 years

**Fig. 1.**
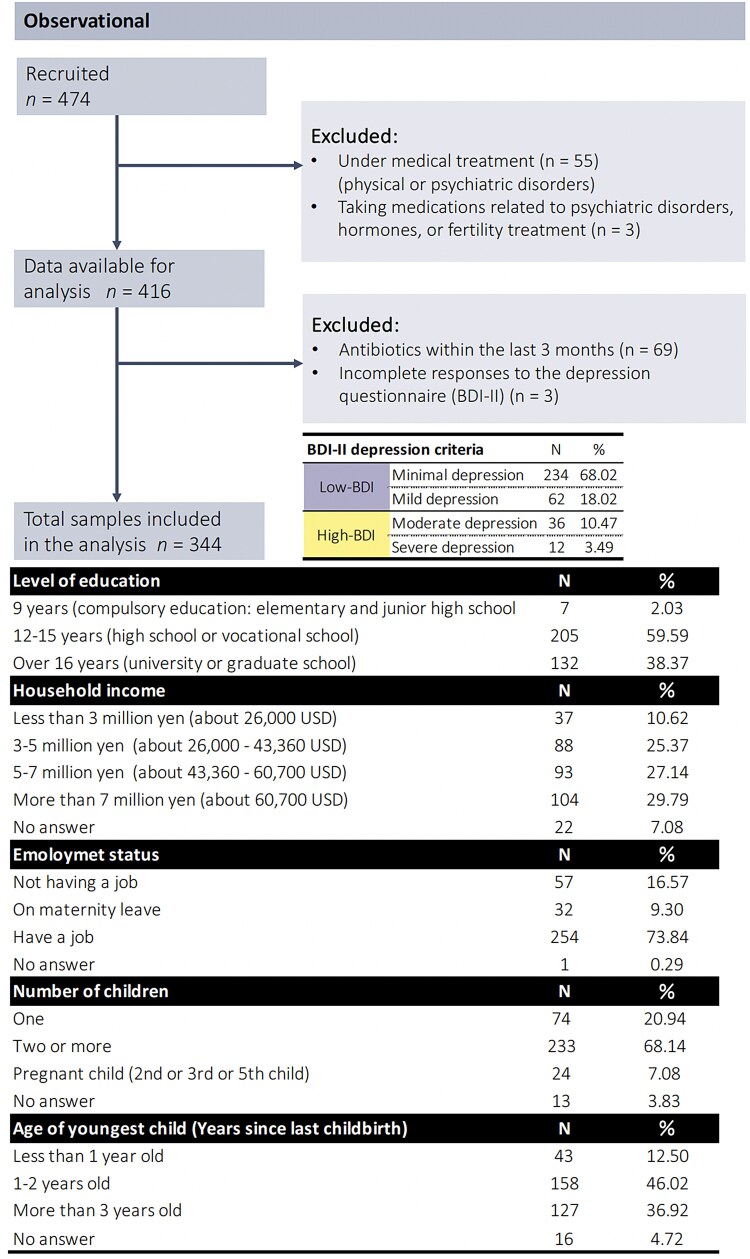
Consort diagram with characteristics of the data pertaining to the analyzed mothers. We analyzed data pertaining to 344 mothers. None of the mothers reported a physical or psychological illness under medical treatment, and none had used antimicrobials within the previous 3 months. We evaluated their levels of depression using the BDI-II. Of the total participants, 296 were classified as having a low-BDI score, and 48 had a BDI score. All of the participants had completed at least 9 years of education. In 2019, the average household income of Japan's child-rearing generation was 7,459,000 yen (about $64,700 US) (41); thus, as shown in this figure, >60% of participants had below-average annual household incomes. The majority of mothers were employed, had two or more children, and had undergone childbirth a few years ago.

The participants who were categorized as having moderate or severe levels of depression were classified into the high-BDI group, following the treatment guidelines of the Japanese Society of Mood Disorders, which recommend medical intervention for individuals scoring above 20 points on the BDI-II due to the increased urgency of addressing their condition. The participants with minimal or mild depression, scoring <20 on the BDI-II, were grouped and taken as the low-BDI group. The results showed that 48 of the 344 mothers (13.95%) were in the high-BDI group, although none of the participants had been diagnosed with any physical or psychological condition (Fig. 1).

**Fig. 2.**
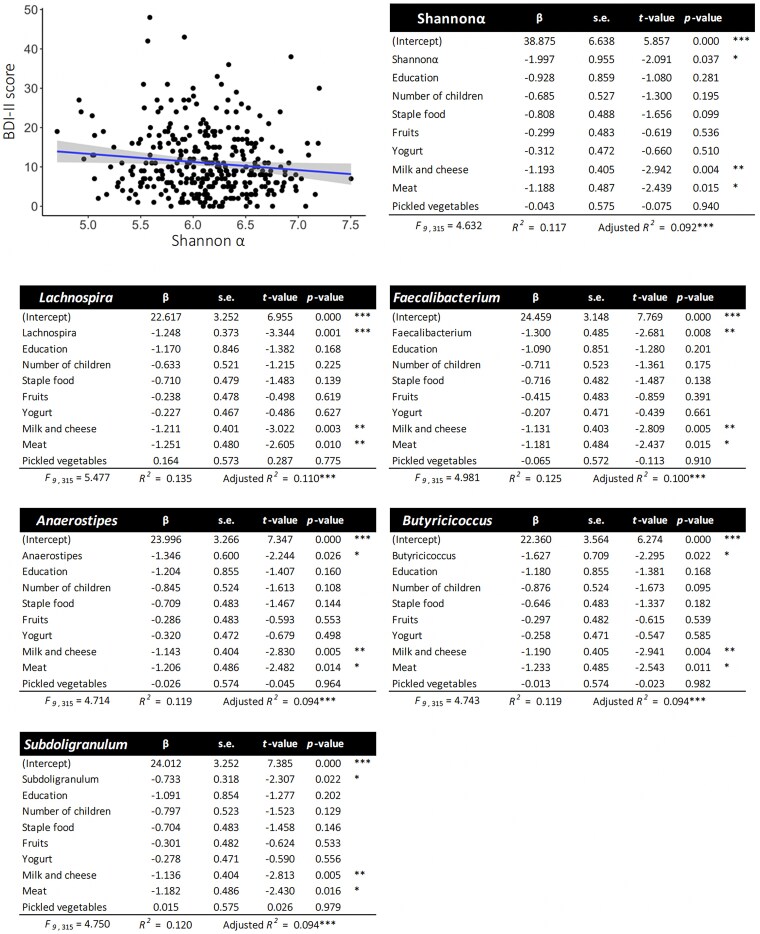
Relationships between intestinal microbiota and depression scores (BDI-II) using multiple regression analysis with covariates. The dependent variable was depression (BDI-II score), the objective variable was intestinal microbiota, and the covariates were level of education, number of children, and mothers' eating habits (frequencies per week). Scatter plots depict the association between intestinal microbiota diversity (Shannon *α*, *x*-axis) and depression scores (BDI-II score, *y*-axis) with 95% CIs. *β*, standardized regression coefficient; s.e., standard error; Education, level of education; Yogurt, a yogurt and lactic acid bacteria beverage. *** *P* < 0.001; **P < 0.01; *P < 0.05.

**Fig. 3.**
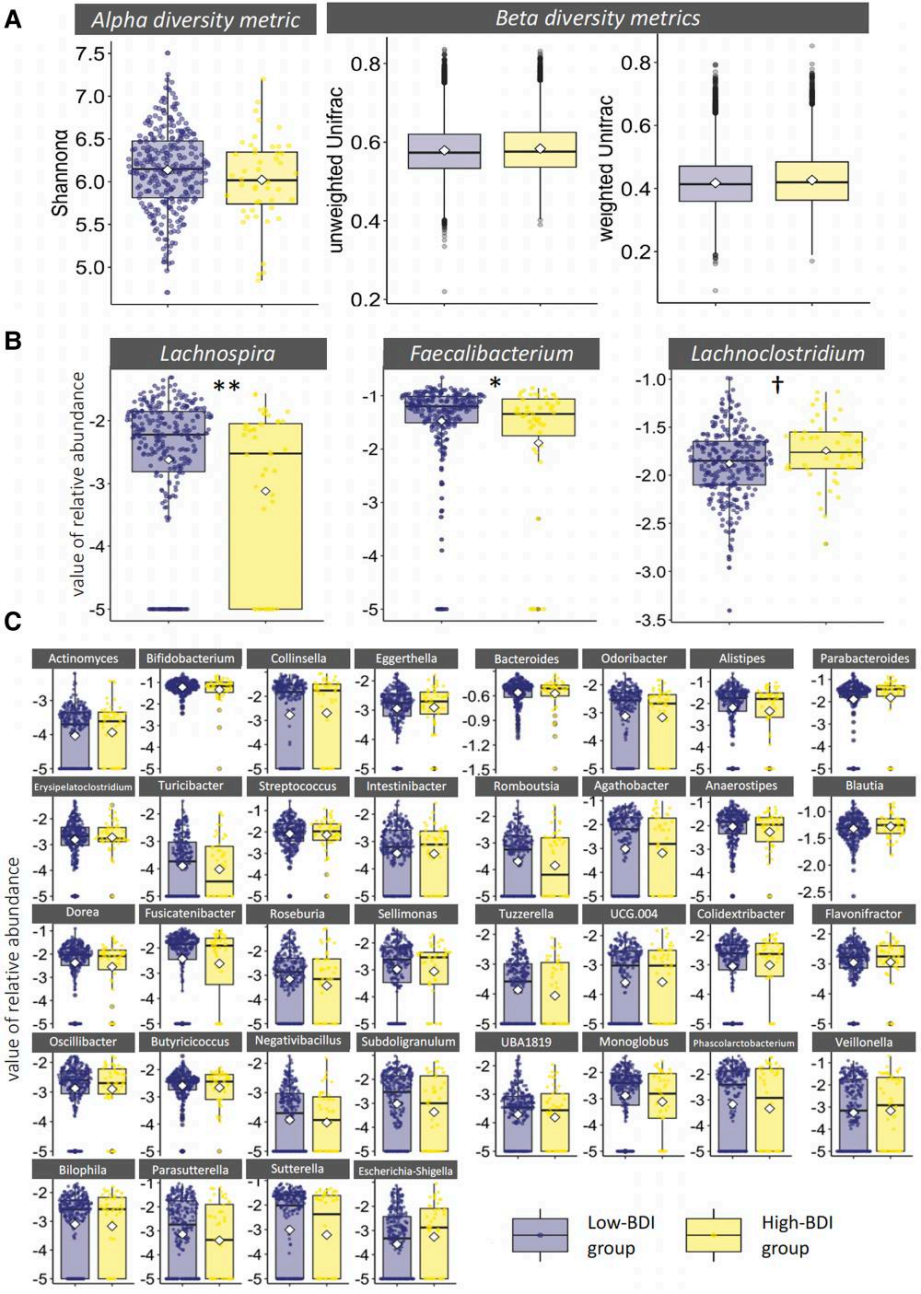
Comparisons between the high- and low-BDI groups using the Mann–Whitney *U* test and ANCOVA. A) Results of the alpha and beta diversity metrics of intestinal microbiota; B) significant results for the RA of genera; and C) nonsignificant results for the RA of genera. UCG.004, Lachnospiraceae_UCG-004. The *x*- and *y*-axes represent groups (high- and low-BDI) and the value of intestinal microbiota, respectively; specifically, they represent the RA of the prevalent microbiota, represented by log10 (*X* + 0.00001). **P and *q*  *<* 0.005 derived using Mann–Whitney *U* test, significant after adjusting for covariates via ANCOVA; *P and *q* < 0.05 derived using Mann–Whitney *U* test but nonsignificant after adjusting for covariates; †*P* < 0.05 and *q* < 0.10 but nonsignificant after adjusting for covariates.

A linear relationship was found between depressive mood (BDI-II score) and intestinal microbiota. The alpha diversity (Shannon *α*) of the intestinal microbiota, especially its relative abundance (RA) of *Anaerostipes*, *Lachnospira*, *Butyricicoccus*, *Faecalibacterium*, and *Subdoligranulu*, was negatively related to depressive mood using Pearson's correlation analysis (*P* < 0.05, *q* < 0.1; Table S1). These relationships remained significant after controlling for covariates such as the level of education, the total number of children, and the dietary habits of mothers who have multiple regression analyses (Fig. 2). In addition, we compared the intestinal microbiota between the high- and low-BDI groups using the Mann–Whitney *U* test. The alpha and beta diversity of the intestinal microbiota showed no significant differences (Shannon *α*; *P* = 0.15, *q* = 0.24, weighted UniFrac distance; *t* = 1.16, *P* = 0.25, unweighted UniFrac distance; *t* = 1.17, *P* = 0.18; Fig. 3). However, the high-BDI group featured greater RA for *Lachnoclostridium* and less RA for *Lachnospira* and *Faecalibacterium* than the low-BDI group (*P* = 0.01, *q* = 0.04; Fig. 3, Table S2). A significant group difference was seen for *Lachnospira* when adjusted for covariates (i.e. level of education, total number of children, and dietary habits) using analysis of covariance (ANCOVA; Table S3). The values for *Faecalibacterium* and *Lachnoclostridium* were nonsignificant after adjusting for covariates.

Using the Mann–Whitney *U* test, we compared the physical conditions, dietary habits, and intestinal microbiota between the high- and low-BDI groups and found that mothers in the high-BDI group had lower subjective sleep-quality ratings and worse MDPS scores than those in the low-BDI group (*P* < 0.01, *q* < 0.01; Table 1). The frequency of the consumption of milk and cheese, yogurt and lactic fermenting beverages, and pickled vegetables was lower in the high-BDI group than in the low-BDI group (*P* < 0.05, *q* < 0.05; Table 1).

**Table 1.** Statistical comparisons of the mother's characteristics, physiological condition, and dietary intake between the high- and low-BDI groups, assessed using the Mann–Whitney *U* test.

		All participants (*n* = 344)		Low-BDI group (*n* = 296)		High-BDI group (*n* = 48)		Mann–Whitney *U* test (high- vs. low-BDI group)		
		Mean	SD	Mean	SD	Mean	SD	*W*	*P*-value	*q*-value
Psychological and physical indices	BDI	11.01	8.16	8.59	5.15	26.78	6.40	0.00	0.000**	0.000**
	Mother's age	34.63	4.78	35.02	4.57	32.23	5.40	8,837.00	0.002**	0.007**
	Level of education	1.36	0.52	1.40	0.53	1.15	0.41	8,862.00	0.001**	0.005**
	Household income	1.82	1.01	1.86	0.97	1.60	1.23	6,925.00	0.213	0.302
	Number of children	2.02	0.83	2.05	0.84	1.81	0.77	7,774.00	0.049*	0.093^a^
	Age of youngest child	1.26	0.67	1.27	0.66	1.18	0.75	6,739.50	0.489	0.370
	Employment status	1.57	0.76	1.59	0.75	1.46	0.85	7,578.50	0.308	0.312
	MDPS total score	14.66	5.69	13.72	5.29	20.46	4.53	2,306.00	0.000**	0.000**
	Physical activity index (MDPS_PAI)	3.08	1.53	2.85	1.45	4.50	1.15	2,719.50	0.000**	0.000**
	Somatic disorders index (MDPS_SDI)	0.98	1.22	0.84	1.12	1.79	1.52	4,429.50	0.000**	0.000**
	Hormone activity index (MDPS_HAI)	4.25	1.74	4.04	1.70	5.52	1.38	3,727.50	0.000**	0.000**
	Microvascular disorders index (MDPS_MDI)	3.63	2.00	3.46	1.94	4.69	2.00	4,658.00	0.000**	0.000**
	Meteoropathy related index (MDPS_MRI)	2.72	1.65	2.52	1.58	3.96	1.54	3,735.50	0.000**	0.000**
	Sleeping time	6.77	1.09	6.79	1.07	6.62	1.21	7,734.00	0.289	0.312
	Sleeping quality	1.91	0.60	1.97	0.58	1.54	0.58	9,658.00	0.000**	0.000**
Dietary intake	Staple food	4.51	0.92	4.53	0.92	4.33	0.91	8,244.50	0.025*	0.060^a^
	Unrefined grain	1.40	0.94	1.42	0.96	1.29	0.85	7,623.00	0.278	0.312
	Root vegetable	3.29	1.05	3.28	1.04	3.35	1.10	6,750.00	0.565	0.370
	Green and yellow vegetables	3.07	1.05	3.07	1.03	3.06	1.17	7,206.50	0.867	0.431
	Light-colored vegetables	3.22	1.03	3.20	1.01	3.31	1.11	6,698.50	0.510	0.370
	Fruits	2.64	1.06	2.66	1.05	2.52	1.13	7,788.50	0.262	0.312
	Meat	3.51	0.97	3.54	0.94	3.31	1.11	7,840.00	0.228	0.311
	Fish and shellfish	2.61	0.86	2.61	0.84	2.60	0.96	7,221.50	0.843	0.431
	Egg	3.02	0.88	3.02	0.87	3.02	0.96	7,210.50	0.860	0.431
	Milk and cheese	2.97	1.18	3.03	1.16	2.54	1.20	8,712.50	0.009**	0.027*
	Yogurt and lactic fermenting beverage	2.31	1.08	2.36	1.09	1.96	0.90	8,435.00	0.028*	0.063^a^
	Soy product	2.60	0.93	2.61	0.92	2.54	1.03	7,464.50	0.545	0.370
	Natto	1.90	0.89	1.89	0.88	2.00	0.95	6,656.00	0.451	0.370
	Pickled vegetables	1.69	0.76	1.71	0.75	1.54	0.80	8,241.00	0.049*	0.093^a^
	Seaweed	2.10	0.77	2.10	0.75	2.06	0.91	7,572.50	0.403	0.370
	Mushroom	2.58	0.86	2.58	0.86	2.52	0.92	7,396.50	0.622	0.379
	Snack	3.45	1.01	3.47	0.96	3.33	1.29	7,344.00	0.689	0.398
	Sugary drink	2.87	1.20	2.85	1.19	2.98	1.26	6,694.50	0.509	0.370

^**^ *P* or *q* < 0.01.

^*^ *P* or *q* < 0.05.

^a^ *q* < 0.10.

### Dietary patterns' effects on depressive moods, physical conditions, and intestinal microbiota

We identified two main dietary patterns using factor analysis (Table 2). The first was a vegetable and meat dietary (VMD) pattern that represented high intakes of vegetables (i.e. green and yellow vegetables, root vegetables, and light-colored vegetables) and meat, along with fish and shellfish. The second factor was termed a soy and fermented food dietary (SFD) pattern, which represented high intakes of soy products, natto, yogurt, and lactic fermenting beverages. SFD was characterized by high intakes of seaweed, mushrooms, fruits, and unrefined grains. Both dietary patterns accounted for 17 and 11%, respectively, of the variance in dietary intakes and explained 28% of the variability.

**Table 2.** Factor loading matrix for major dietary patterns that were identified via factor analysis.

	Vegetable and meat dietary pattern (VMD)	Soy and fermented food dietary pattern (SFD)
Green and yellow vegetables	**0.85**	−0.01
Root vegetable	**0**.**80**	0.01
Light-colored vegetables	**0**.**72**	0.00
Meat	**0**.**52**	0.07
Staple food	**0**.**47**	−0.18
Fish and shellfish	**0**.**39**	0.29
Snack	0.12	0.03
Soy product	0.00	**0**.**78**
Natto	−0.12	**0**.**48**
Yogurt and lactic fermenting beverage	−0.07	**0**.**45**
Seaweed	0.19	**0**.**39**
Mushroom	**0**.**30**	**0**.**38**
Fruits	0.27	**0**.**32**
Unrefined grain	−0.07	**0**.**31**
Milk and cheese	0.17	0.28
Egg	0.23	0.28
Pickled vegetables	−0.05	0.22
Sugary drink	0.09	−0.21

Factor loadings with an absolute value of ≥ 0.30, indicating a substantial contribution to the factor, are shown in bold.

We present participant characteristics according to the high and low scores for dietary patterns with the results of a t test (Table S4). The high-VMD group exhibited a higher frequency of consumption for the 13 food categories (*P* < 0.05; *q* < 0.10), with the exception of unrefined grain, natto, pickled vegetables, and sugary drinks. In contrast, the high-SFD group showed a higher frequency of consumption of the 16 food categories (*P* < 0.05, *q* < 0.10), with the exception of staple food and snacks. These results indicate that the high-SFD group consumed more vegetables, meat, and fish than did the low-SFD group; further, the high-SFD group consumed soy products, fermented foods, seaweed, and mushrooms more frequently, which is representative of the high-quality and healthy Japanese food patterns reported previously (35, 37).

**Fig. 4.**
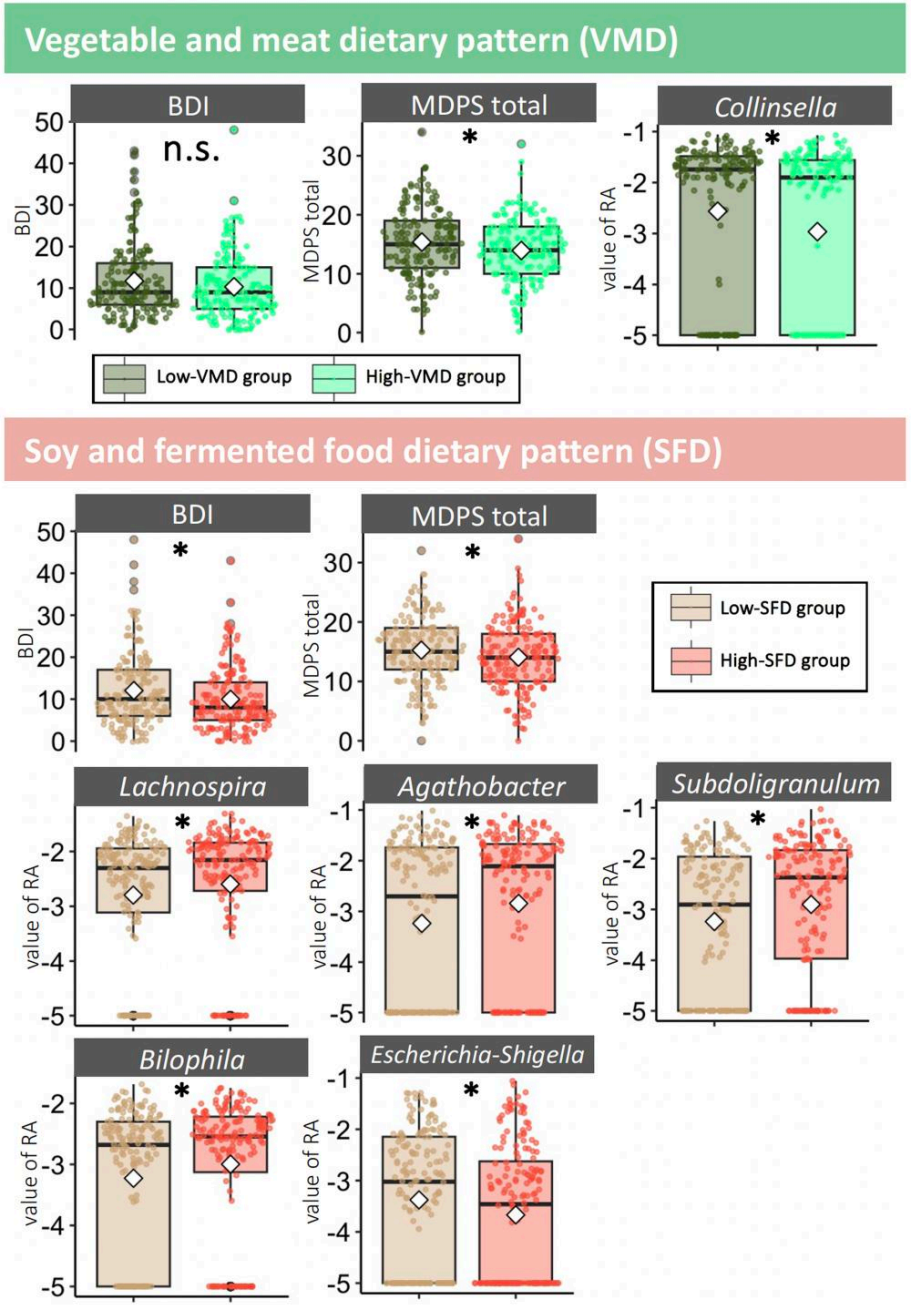
Comparison between the scores of the high and low dietary pattern groups (VMD and SFD) using ANCOVA. The results for the depression and physical condition scores (MDPS total), as well as gut microbiota, are significant at *P < 0.05 via ANCOVA, adjusted for covariates level of education, total number of children, and employment status. The *x*-axis represents the high or low group for each dietary pattern (i.e. VMD or SFD), and the *y*-axis represents the BDI-UU and MDPS total scores or the value of the RA of the intestinal microbiota, represented by log10 (*X* + 0.00001).

To investigate the effects of the VMD and SFD dietary patterns on depressive mood, physical condition, and intestinal microbiota, we compared the high- and low-scoring groups for each dietary pattern, using the median as the cutoff (Fig. 4). With respect to the effects of VMD and SFD on depressive mood and physical condition, we performed ANCOVA on the covariates (i.e. level of education, total number of children, and employment status). For the VMD effect, no significant differences were found in the depressive mood between the high- and low-VMD groups (*F*_1,319_ = 2.91, *P* = 0.09). However, in terms of physical condition, the low-VMD group exhibited higher total scores for MDPS than the high-VMD group (*F*_1,325_ = 5.68, *P* = 0.02). Meanwhile, with respect to the effects of the SFD, there were significant group differences between the high- and low-SFD groups in terms of depressive mood (*F*_1,319_ = 4.67, *P* = 0.02) and physical conditions in the total MDPS score (*F*_1,325_ = 3.91, *P* = 0.05). The low-SFD group had a higher depressive mood and worse physical condition. For intestinal microbiota, the high-VMD group showed significantly greater RA for *Collincella* than the low-VMD group (*F*_1,325_ = 4.65, *P* = 0.03). However, the high-SFD group had significantly greater RA for *Agathobacter* (*F*_1,325_ = 5.51, *P* = 0.02), *Lachnospira* (*F*_1,325_ = 4.07, *P* = 0.04), and *Subdoligranulum* (*F*_1,325_ = 4.59, *P* = 0.03), *Bilophila* (*F*_1,325_ = 5.05, *P* = 0.03), and lower RA for *Escherichia* and *Shigella* (*F*_1,325_ = 4.07, *P* = 0.04) than the low-VMD group. All of these results were adjusted in terms of covariation by ANCOVA. See Tables S5 and S6 for detailed statistics and null results.

## Discussion

This study comprehensively investigated the intestinal microbiota, physical condition, and dietary factors of mothers in Japan caring for children aged 0–4 years in association with their depressive mood. We found that the intestinal microbiota, in particular those producing short-chain fatty acids (e.g. *Lachnospira*, *Faecalibacterium*, and *Subdoligranulum*), were associated with depressive mood. Clinical studies of patients who have depressive disorder have found that they often have sleep disturbances (e.g. insomnia and hypersomnia), anorexia, and negative physical conditions (e.g. malaise, headache, palpitations, dizziness, and tinnitus) (13–16). This present showed that, even among mothers in a healthy population, those who had a high-depression score had worse sleep quality, worse physical condition, and lower consumption of staple foods, milk, cheese, yogurt, and lactic acid bacteria beverages relative to mothers who had low depressive scores. We also found that Japanese-style eating habits are beneficial to mental and physical health, including the growth of intestinal microbiota (e.g. *Agathobacter*, *Lachnospira*, and *Subdoligranulum*).

Our results showed that ∼14% of participants potentially had severe depression based on their BDI-II scores, although all of the participants were considered to be part of a healthy population with no diagnosis of or treatment for a clinical psychiatric or physical illness. As assessed by MDPS scores (40), mothers with high-depression scores were in poorer physical condition, especially with respect to their hormonal balance, digestive function, and blood circulation. In particular, mothers with high-depression scores showed notably reduced sleep quality and less frequent consumption of staple foods than mothers in the low-depression score group. These results support the application of the DSM-V diagnostic criteria for depression, insomnia, and anorexia.

For the intestinal microbiota, we found that its diversity (i.e. Shannon *α*) was negatively related to depression scores (BDI-II), in particular those for *Lachnospira*, *Faecalibacterium*, *Anaerostipes*, *Butyricicoccus*, and *Subdoligranulum*; these associations were significant even after the mother's level of education, total number of children, and diet were taken into account. That is, mothers who had greater intestinal microbiota exhibited lower BDI depression scores. The comparison of high- and low-depression scores in mothers also revealed a significantly different RA for the *Lachnospira.* Notably, all bacteria that were associated with depression in this study produced butyrate (42–44). The most recent research and systematic reviews have found lower rates of butyrate-producing bacteria in depressed and postpartum-depressed patients (21–23). SCFAs are produced by gut microbiota-induced fermentation of dietary fiber. Among SCFAs, butyrate-producing bacteria in particular have several beneficial effects on the intestinal environment, including the improvement of the immune function of the intestinal mucosa (42, 43, 45). Our results are consistent with those of previous studies, which suggest that having lower rates of butyrate-producing bacteria could increase depression among mothers in the healthy population.

In addition, we found that high-quality, healthy Japanese diet patterns could benefit mental and physical health, including improvements to the intestinal microbiota. This study identified two main dietary patterns, namely, VMD (high intake of vegetables and meat/fish) and FMD (high intake of soybeans, fermented foods, seaweed, and mushrooms), along with the intake of vegetables and fruits. In particular, FMD showed food components that were similar to the characteristics that were identified in previous studies as typical patterns of a high-quality, healthy Japanese diet (32, 37). In a comparison of mothers having high and low FMD, the high-FMD group had lower depressive mood and better physical condition, as assessed by MDPS scores. Furthermore, mothers with higher FMD scores had greater RA for *Agathobacter*, *Lachnospira*, *Subdoligranulum*, *and Bilophila* and lower RA for *Escherichia* and *Shigella* than mothers who had low-FMD scores. We observed no effect on depressive mood between the high- and low-VMD groups, and this difference was related to the physical condition of the MDPS. Notably, the active intake of soybeans, fermented foods, seaweed, mushrooms, fruits, and unrefined grains, rather than just taking vegetables, meat, and fish, could be effective in promoting mental and physical health and preventing psychosomatic disorders among mothers raising children in Japan.

*Agathobacter*, *Lachnospira*, and *Subdoligranulum*, which were significantly more prevalent in the high-SFD group, are also short-chain, fatty acid–producing bacteria (42–44), which previous studies associated with depression and anxiety disorders (19, 21, 22). In particular, previous studies have found that *Subdoligranulum* is associated with the quality of diet. For example, those on a western-style, low-fiber diet that is rich in processed meats, fat, sugar, and sodium showed reduced *Subdoligranulum* relative to those on a plant-based, high-fiber diet rich in fruits, vegetables, and whole grains (46). *Subdoligranulum* was found to be increased in individuals with an omega-3-rich diet (47). A meta-analysis has shown that omega-3 fatty acids, specifically eicosapentaenoic acid supplementation, are beneficial for depression (48). Omega-3 fatty acids are abundant in fish, soybeans, walnuts, and vegetable oils. That study found that mothers in a high-FMD group consumed more vegetables, fish, and soy products than those in a low-FMD group. Therefore, this pattern potentially exerted an effect on the depressive mood. Specifically, previous studies reported that foods, such as soy products, seaweed, and mushrooms—which are characteristic of SFD but not VMD—contain abundant antioxidants and have anti-inflammatory properties (49–51). A previous nutritional epidemiology study examined the association between diet and depression in Japan, with a healthy Japanese dietary pattern consisting of food items that were similar to those in the current SFD, associated with high intakes of anti-inflammatory nutrients, including folate; vitamins B, C, E, and D; magnesium; calcium; iron; and dietary chlorine, which was also associated with depression relief (37). Several traditional Japanese fermented foods, using soybeans and rice bran (including seasonings, e.g. soy sauce, miso, sake, and vinegar), contain *koji*. Studies conducted on mice demonstrated that glucosylceramide—a component of *koji*—increases butyric acid bacteria (52), and reports have found that dietary intervention with glucosylceramide from soybeans and rice bran reduces the incidence of cancer (53, 54). In recent years, the consumption of fermented foods has attracted attention in terms of the diet–microbiota–immune system axis. In a study of dietary interventions in adults, a high-fermented food diet steadily increased microbiota diversity and decreased inflammatory markers, whereas high-fiber intake had no effect (55). Moreover, mushrooms naturally produce precursors of vitamin D. A meta-analysis for observational studies and randomized controlled trials indicated that participants who had low levels of vitamin D tended to have a stronger association with depression relative to a control (56). Furthermore, previous studies have shown a positive association between vitamin D concentrations in serum and the diversity of the intestinal microbiota, in which rates of butyric acid bacteria were higher (57). The results of this study show that a healthy Japanese dietary pattern—characterized by a rich intake of soy products, fermented foods, seaweed, and mushrooms, along with fruits and vegetables—is beneficial in terms of nutrients and the gut microbiota associated with butyrate production. The evidence that these dietary patterns are beneficial to gut microbiota and body–mind health, even in healthy mothers, implies that high-quality Japanese dietary patterns and foods contribute to the prebiotic function of improving gut microbiota and helping Japanese mothers recover from mental and physical health problems.

This study had certain limitations. First, it was cross-sectional in nature, so we could not assess intraindividual changes that take place before, during, or after childbirth. Therefore, longitudinal studies including larger samples are required to elucidate the neurophysiological–psychological relationships. Case–controlled studies should incorporate samples from clinical groups with severe levels of depression. It is important to take account of factors related to the mother and child, including those related to the parenting environment and the degree of support. To expand consideration of depressive symptoms for clinical application in the future, detailed validation with shotgun analysis and the direct evaluation of the amounts of metabolites, including SCFA, which were associated with this study, would also be beneficial. Second, it is necessary to identify the direct relationship between physical and mental health and dietary habits. This study used an 18-item questionnaire to identify the frequency of food intake. The results indicated that the SFD has a potential influence on the microbiota and physical and mental health. However, our dietary data do not distinguish between rice and bread intake, types of meat (processed or not), types of fish, or types of processed food. Thus, we cannot rule out the possibility that differences in intakes of these foods could have influenced our results. For these reasons, future work should verify the results using a detailed and rigorous measurement of dietary data and performing nutritional calculations. For future studies, especially those involving dietary intervention, it will be advantageous to perform shotgun metagenomic analysis to identify the specific species and strains of bacteria. The accumulation of such fundamental research can promote the development of dietary methodologies contributing to the prevention and promotion of physical and mental health. Third, unmeasured confounders and sample selection bias are important issues. We adjusted for covariates that are significantly associated with depression and dietary patterns in the available variables in the questionnaire, including age, level of education, household income, employment status, total number of children, age of the youngest child (years since last birth), and food intake. However, we did not measure other related factors (e.g. marital status, body composition, exercise habits, and family history of mental health). Future studies should assess these factors to study the abovementioned associations to improve the quality of studies in this field. Finally, while the characteristics of the Japanese healthy dietary patterns were associated with depression, physical condition, and intestinal microbiota, the question of whether the current results are specific to the data obtained in Japan or are common to the rest of the world is crucial. For example, Mediterranean dietary patterns—which are similar to those of healthy Japanese diets in terms of their high intakes of vegetables, fruits, and beans—were associated with a lower incidence of depression (58). Thus, future studies should explore the accumulation of further international and Japanese comparative studies.

This study showed that the intestinal microbiota of short-chain, fatty acid–producing bacteria are associated with a high degree of depressive mood in healthy mothers who are caring for young children in Japan. Additionally, high-depression mothers exhibited worse sleep quality, physical condition, and dietary habits. Our results indicate that the following dietary characteristics can benefit mind–body health: a high-quality, healthy Japanese-style diet, especially including the intake of fermented foods; a high-fiber, plant-based diet, including not only vegetables, meat, and fish but also soy products, mushrooms, seaweed, and fruits, as well as yogurt and lactic acid bacteria beverages, which are thought to increase the rate of butyrate-producing bacteria. In recent years, nutritional psychiatry has also begun to obtain observational and efficacy data that support the role of healthy dietary patterns in the development of depression and the management of its symptoms. The evaluation and support of mothers who have depression are important in terms of their intestinal microbiota, physical condition, and dietary habits, based on the gut–brain axis rather than solely from a psychological viewpoint. Furthermore, we suggest that nonpharmacological methods, such as dietary intervention utilizing gut microbiota, not only in the perinatal period but also in the long term, could effectively prevent and ameliorate the onset of depressive disorders in healthy mothers who have not yet developed depression. Because research on women's mental and physical health has not yet been extensively conducted (59), this study can highlight the need for further research and promote qualitative improvement in mental health care.

## Materials and methods

### Participants and procedures

As part of the Japanese research project The Principle of Human Social Brain-Mind Development, this study was conducted to eventually create a database of the intestinal microbiota of >1,300 mother–child pairs in Japan and investigate the relationships between the microbiota and infant emotional and cognitive development. The participants were recruited from throughout Japan through nursery schools that agreed to cooperate in the study and using intestinal microbiota data collected in January and February 2021. The Medical Ethics Committee of Kyoto University (no. R2624) approved this study, and it was registered in the UMIN system (UMIN000042508). We obtained written informed consent from each participant and collected a stool sample. Respondents completed the study questionnaire. All participants were Asian women, as per the classification of the US Office of Management and Budget (60). The informed consent document outlined the eligibility and exclusion criteria, and only those who agreed to participate and were eligible were included. No specific identification was provided in the questionnaire. The eligibility criteria were as follows: mothers with children (aged 2 months to 6 years) attending preschool in Japan who are Japanese citizens and primarily reside in Japan. The exclusion criteria were mothers who were hospitalized due to serious psychiatric or physical disorders, infants at risk for severe developmental disorders—such as chromosomal abnormalities—and mothers who could not understand the study content or respond adequately to the questionnaire.

Figure 1 shows the consort diagram and the participants' characteristics. We recruited 474 participants but excluded those undergoing treatment for psychiatric and/or physical diseases (*n* = 55) and taking medication related to depression or female hormones (*n* = 3). Of the 416 participants whose data were available for analysis, we excluded those who had used antimicrobials within 3 months of the time of study entry (*n* = 69) and those with incomplete responses to the depression questionnaire (BDI-II) (*n* = 3). Finally, we analyzed data from 344 participants (mean age = 34.63, SD = 4.78, range: 21–47 years) The data analyzed were from participants without psychiatric or physical disorders during the period of data measurement (see Fig. 1 for participant characteristics). We confirmed the appropriate sample size a priori using G*Power. Assuming that using ANCOVA, adjusted for covariates, would require the largest sample size in this study, the study set the number of predictors to a maximum of 10, the effect size to a moderate 0.25 or a large 0.40, and power to 0.80. It was estimated that a sample size of 162 and 400 participants would be required for large- and medium-effect sizes, respectively.

### Questionnaires

We used questionnaires to measure depression, physical condition, dietary habits, and socioeconomic status (Table S7). To assess the severity of the depressive symptoms, we used the BDI-II, including 21 items rated on a 4-point scale (38), based on the diagnostic criteria of the DSM-IV, which has been validated and standardized in Japanese (39). Following the treatment guidelines of the Japanese Society of Mood Disorders, we considered 20 points as the cutoff for depression. The high-BDI group in this study was a subclinical sample without diagnosed depression that may require clinical treatment. In other words, this group is at a high risk of depression. The low-BDI group, in contrast, was healthy postpartum women at low risk of depression.

To assess physical condition, we used the MDPS (40), based on the Oriental Medicine Criteria for Physical Pathology in women. The MDPS consists of 17 items, rated on a 3-point scale, along the following five subscales: (i) the physical activity index, on which higher scores indicate physical inactivity (e.g. fatigue and physical lethargy); (ii) the somatic disorders index on which higher scores indicate high physical depression symptoms (e.g. anorexia and bloating); (iii) the hormone activity index, on which higher scores indicate poor (female) hormone function (e.g. coldness, dizziness, and dry skin); (iv) the microvascular disorders index, on which higher scores indicate impaired microcirculation related to (female) hormone function (e.g. skin pigmentation and rough skin); and (v) the meteoropathy index, on which higher scores indicate impaired water metabolism and meteorological disease (e.g. swelling and headaches due to bad weather).

We measured dietary and lifestyle habits with the Mykinso Pro questionnaire, which includes questions on lifestyle (e.g. smoking, drinking, sleep, and defecation), dietary intake, and physical disease (e.g. medical treatment history and medication). This questionnaire was developed by Cykinso Inc., a Japanese provider of screening services for intestinal microbiota. For sleep duration and quality, the participants reported their average number of hours of sleep per day and their sleep quality on a 3-point scale (3 = good; 2 = fine; 1 = bad). To assess diet, the questionnaire includes 18 components: staple foods (e.g. rice, bread, and noodles), unrefined grains, root vegetables, green and yellow vegetables, light-colored vegetables, fruits, meat, fish and shellfish, eggs, milk and cheese, yogurt and lactic acid bacteria beverages, soybeans and soybean-derived foods, natto (fermented soybeans), seaweed, pickles, mushrooms, snacks, and sugary drinks. The questionnaire asked participants how frequently they consumed each of these foods during the week before stool collection on the following 6-point scale: 1 = did not eat; 2 = 1–3 times/week; 3 = 4–6 times/week; 4 = once/day; 5 = twice or more/day. The questionnaire also collected demographic information (e.g. age, educational background, and employment status), as well as socioeconomic status.

### Identification and evaluation of dietary patterns

We performed factor analysis in relation to the 18 food and beverage categories to derive dietary patterns. The factors were extracted using maximum likelihood and were rotated by the oblimin rotation (oblique rotation). We considered eigenvalues, the Velicer minimum average partial (MAP), the Bayesian information criterion (BIC), a screen test, and the interpretability of the factors to identify the number of factors to be retained. These factors satisfied the criteria of eigenvalues >1 and MAP and BIC of a factor number of two. The scree plot showed a substantial decrease for the second factor and remained similar for the third factor. Therefore, we retained two factors. Dietary patterns were named according to the food items, which displayed high loadings (absolute values) for two factors. The study calculated the factor scores for each dietary pattern and each individual through summation of the intakes of food items, weighted by their factor loadings. The factor scores were categorized into two groups (high and low), where the median was the cutoff.

### Intestinal microbiome analysis

#### Fecal sample collection and DNA extraction

Fecal samples were collected using Mykinso fecal collection kits that contained guanidine thiocyanate solution (Cykinso, Tokyo, Japan), were transported at ambient temperature, and were stored at 4 °C. DNA extraction was performed using an automated DNA extraction machine (GENE PREP STAR PI-480; Kurabo Industries Ltd, Osaka, Japan), following the manufacturer's protocol. DNA extraction and 16S rRNA sequencing were performed at Cykinso, Inc. (Tokyo, Japan), and shotgun sequencing was conducted at the Genome Information Research Center of the Research Institute for Microbial Diseases, Osaka University (Japan).

#### 16S rRNA gene sequencing

The detailed sequencing methods have been described elsewhere (61). Briefly, amplicons of the V1V2 region were prepared using the forward primer (16S_27Fmod: TCG TCG GCA GCG TCA GAT GTG TAT AAG AGA CAG AGR GTT TGA TYM TGG CTC AG) and the reverse primer (16S_338R: GTC TCG TGG GCT CGG AGA TGT GTA TAA GAG ACA GTG CTG CCT CCC GTA GGA GT). The libraries were sequenced in a 250 bp paired-end run using the MiSeq Reagent Kit v2 (Illumina; 500 cycles). Each of the samples was sequenced to a depth that ensured that a minimum of 10,000 reads remained following filtering, and the samples were sequenced again if they did not meet this threshold.

#### Bioinformatics analysis

Data processing and assignment were performed using the QIIME2 pipeline (version 2020.8) (62) following these steps: (i) joining paired-end reads, filtering, and denoising using DADA2 (63) and (ii) assigning taxonomic classifications for amplicon sequence variants using the naïve Bayes classifier in QIIME2. The classifier was trained using arts-SILVA (64), a simplifier for the SILVA (65) 138 16S rRNA taxonomy dataset, which was designed to enhance comprehensibility by providing refinements to taxonomic assignments and curating misentries in the SILVA database. In particular, arts-SILVA omits labels that feature minimal information, corrects duplicate entries, and excludes irrelevant taxa, such as the “human metagenome,” using manual inspection. It only assigns a consensus taxonomy to each cluster when a 100% agreement exists among the assigned taxa and then eradicates the label “ambiguous taxa” to ensure a clear and concise taxonomy for analysis. We performed all data manipulation, analyses, and graphics using R and RStudio (versions 3.5.1 and 1.1.456, respectively), using the R package qiime2R and microbiome R for all analyses. We used the R packages tidyMicro (version 1.48) and ggplot2 for the visualizations. To evaluate the diversity of the intestinal microbiota, we measured alpha diversity (at 10,000 reads per sample) at the ASV level using the Shannon index and beta diversity using unweighted and weighted UniFrac distances. The unweighted UniFrac distance indicates the beta diversity that incorporates the presence/absence of taxa; the weighted UniFrac distance indicates the beta diversity that incorporates the RA of the taxa. Additionally, based on previous studies, we analyzed the prevalent microbiota's composition at the set of genus-level bacterial groups that are shared by at least 50% of the participants in RA of at least 0.01% (66); we calculated the RA as the number of sequencing reads for each taxon in a sample that was standardized using the total number of sequences that were generated for that sample. In the analyses, we only included taxa that were present in at least one sample, and we aggregated sequence counts for taxa that did not meet the requirements into an “other” category. We applied these filters at the phylum, family, and genus levels, leaving only sequence counts that we could not classify at the taxonomic level of interest as unclassified counts on the lowest level possible. See Table S8 for more details of the core microbiome that is detected in this study.

## Statistical analysis

Using R and RStudio (versions 4.2.1 and 2022.12.0, respectively), we performed statistical analyses and visualizations for both studies. Correction for multiple testing (*q*-value estimation using the R package Bioconductor version 3.15 with R and RStudio [versions 4.2.1 and 1.4.1106, respectively]) was applied, and significance was defined as *P* < 0.05 and *q* < 0.1. All of the statistical tests used were two-tailed.

To select the covariates to be adjusted, we checked correlations between depression scores (i.e. BDI-II) and the demographic information (i.e. age, level of education, household income, total number of children, age of the youngest child, and employment status) with 18 dietary items (Table S1). We further compared these indices between the high- and low-BDI groups using the Mann–Whitney *U* test to identify the covariates that were associated with depression (Table 1). The study presents the following characteristics as candidates for covariates: mother's age, level of education, and total number of children. However, we also observed a significant correlation between age and level of education (*r* = 0.17, *P* = 0.002). The sample was limited to mothers who were raising children aged 0–4 years; this age-based population was considerably more controlled than those investigated in previous studies. In addition, the level of education was potentially related to the knowledge of nutrition and mental illness, as well as to health consciousness and food choices. Taking possible multicollinearity into account, we used the level of education and the total number of children as covariates. Finally, we adjusted the level of education (measured as 0: compulsory education, 1: high school or vocational school, 2: university or graduate school), number of children (one to five children), and eating habits. (i.e. staple food, fruits, meat, milk and cheese, yogurt and lactic fermentation beverage, and pickled vegetables; 1 = did not eat; 2 = 1–3 times/week; 3 = 4–6 times/week; 4 = once/day; 5 = twice or more/day) as covariates, while analyzing the association between depression and intestinal microbiota. The analytical results of the effects of dietary patterns on depression and intestinal microbiota highlighted a new and significant group difference in employment status between high- and low-BDI groups adopting the t test (Table S4). The following variables were adjusted as covariates for the analysis of the impact of dietary patterns, including items related to depression: level of education, total number of children (one to five children), and employment status measured as 0: not having a job, 1: having a job but on maternity leave, and 2: having a job.

We performed Pearson's correlation analysis between the depression score and the index of the microbiota. Then, we performed multiple regression analysis to investigate the linear relationships between intestinal microbiota and depression scores on the BDI-II using forced entry for all covariates, as follows. We used the depression score as the dependent variable. For the independent variables, we entered indices of intestinal microbiota that were significantly related to the depression score, taking the following indices as covariates: mother's educational background, total number of children, and dietary habits.

Furthermore, we used the Mann–Whitney *U* test to compare the high- and low-BDI groups with respect to physical conditions (i.e. sleep duration, sleep quality, and MDPS scores), dietary habits, and intestinal microbiota (i.e. alpha diversity and prevalent microbiota). With respect to the group comparison of the beta diversity of the intestinal microbiota, we conducted PERMANOVA. Additionally, we conducted ANCOVA to examine whether the results obtained by comparing the high- and low-BDI groups remained significant when adjusting for covariates.

To identify the effects of dietary patterns on depression, physical condition, and intestinal microbiota, we compared the high and low groups of each dietary pattern (i.e. VMD and SFD) using ANCOVA. For the dependent variables, we entered the score for depression (BDI-II) and total MDPS or the indices of intestinal microbiota (i.e. alpha diversity and prevalent microbiota). For the independent variables, we entered the dietary pattern group (0: low group; 1: high group) and the following covariates: level of education, total number of children, and employment status.

## Supplementary Material

pgaf169_Supplementary_Data

## Data Availability

All sequencing data have been deposited in the NCBI Sequence Read Archive under the project and are publicly available. The BioProject accession numbers are as follows: PRJNA844514 (16S rRNA gene sequencing). All questionnaire data are available from the Figshare database (DOI: 10.6084/m9.figshare.25719909).
