# Supplementary material for: Association of short-chain fatty acid–producing gut microbiota and dietary habits with maternal depression in a subclinical population
Source: PNAS Nexus. 2025 Sep 2;4(9):pgaf169. doi: 10.1093/pnasnexus/pgaf169 (PMC12404299; doi:10.1093/pnasnexus/pgaf169)
Supplement: pgaf169_Supplementary_Data [file pgaf169_supplementary_data.pdf]

Supplementary Information for

Association of Short-Chain Fatty Acid-Producing Gut Microbiota and Dietary Habits with Maternal Depression in a Subclinical Population.

Michiko Matsunaga, Mariko Takeuchi, Satoshi Watanabe, Aya K. Takeda, Keisuke Hagihara, and Masako Myowa.

Correspondence should be addressed to Michiko Matsunaga.
Email: paprika3c5@gmail.com

This PDF file includes:

Content	Page
**Supplementary Table 1.** Correlations between BDI-II Score and Participants' Characteristics, Dietary Intake, and Intestinal Microbiome	2
**Supplementary Table 2.** Statistical Comparisons of Intestinal Microbiomes between the High- and Low-BDI Groups Using the Mann–Whitney U test	3
**Supplementary Table 3.** Statistical Comparisons of the Intestinal Microbiomes between the High- and Low-BDI Groups Using ANCOVA with Covariates	4-7
**Supplementary Table 4.** Statistical Comparisons of Depression, Physical Condition, and Intestinal Microbiome between the High- and Low-VMD Groups Using a T-test	8
**Supplementary Table 5.** Statistical Comparisons of Depression, Physical Condition, and Intestinal Microbiome between High- and Low-VMD Groups via ANCOVA with Covariates	9-11
**Supplementary Table 6.** Statistical Comparisons of Depression, Physical Condition, and Intestinal Microbiome between High- and Low-SFD Groups via ANCOVA with Covariates	12-14
**Supplementary Table 7.** The list of questionnaires and orders	15-17
**Supplementary Table 8.** Genera Prevalent in the Microbiomes in This Study	18

Supplementary Table 1. Correlations between BDI-II Score and Participants' Characteristics, Dietary Intake, and Intestinal Microbiome

Correlation between BDI and characteristics	Pearson's *r*	*p* -value		*q* -value	
Mother's age	-0.12	0.029	*	0.061	†
Level of education	-0.07	0.181		0.118	
Household income	-0.10	0.079		0.089	†
Number of children	-0.09	0.095		0.094	†
Age of youngest child	0.04	0.449		0.205	
Emoloyment status	-0.04	0.428		0.205	

Correlation between BDI and dietary intake	Pearson's *r*	*p* -value		*q* -value	
Staple food	-0.14	0.012	*	0.037	*
Unrefined grain	-0.02	0.749		0.292	
Root vegetable	-0.07	0.231		0.145	
Green and yellow vegetable:	-0.08	0.153		0.118	
Light colored vegetables	-0.07	0.182		0.118	
Fruits	-0.14	0.011	*	0.037	*
Meat	-0.19	0.000	**	0.006	**
Fish and shellfish	-0.06	0.259		0.150	
Egg	-0.09	0.117		0.106	
Milk and cheese	-0.23	0.000	**	0.000	**
Yogurt and lactic fermenting	-0.13	0.021	*	0.058	†
Soy product	-0.07	0.177		0.118	
Natto	0.10	0.078		0.089	
Pickled vegetables	-0.02	0.649		0.257	
Seaweed	-0.11	0.050		0.084	†
Mushroom	-0.10	0.079		0.089	
Snack	-0.11	0.052		0.084	†
Sugary drink	0.03	0.613		0.247	

Correlation between BDI and intesitnal microbiome	Pearson's *r*	*p* -value		*q* -value	
Shannonα	-0.12	0.029	*	0.061	†
Actinomyces	0.09	0.095		0.094	†
Bifidobacterium	-0.07	0.179		0.118	
Collinsella	-0.01	0.865		0.326	
Eggerthella	0.01	0.816		0.313	
Bacteroides	-0.07	0.179		0.118	
Odoribacter	-0.06	0.254		0.150	
Alistipes	-0.10	0.067		0.089	†
Parabacteroides	0.00	0.936		0.336	
Erysipelatoclostridiur	0.06	0.303		0.167	
Turicibacter	-0.04	0.436		0.205	
Streptococcus	-0.10	0.066		0.089	†
Intestinibacter	-0.01	0.897		0.327	
Romboutsia	-0.04	0.463		0.205	
Agathobacter	-0.01	0.887		0.327	
Anaerostipes	-0.11	0.045	*	0.084	†
Blautia	0.00	0.978		0.346	
Dorea	-0.05	0.385		0.198	
Fusicatenibacter	-0.08	0.152		0.118	
Lachnoclostridium	0.09	0.110		0.104	
Lachnospira	-0.18	0.001	**	0.006	**
Roseburia	-0.08	0.145		0.118	
Sellimonas	-0.05	0.399		0.200	
Tuzzerella	-0.03	0.532		0.225	
UCG-004	-0.06	0.300		0.167	
Colidextribacter	-0.05	0.360		0.189	
Flavonifractor	-0.05	0.347		0.187	
Oscillibacter	-0.03	0.590		0.243	
Butyricicoccus	-0.12	0.024	*	0.060	†
Faecalibacterium	-0.15	0.004	**	0.025	*
Negativibacillus	-0.04	0.508		0.221	
Subdoligranulum	-0.15	0.007	**	0.034	*
UBA1819	-0.08	0.143		0.118	
Monoglobus	-0.10	0.076		0.089	†
Phascolarctobacteriu	-0.09	0.090		0.094	†
Veillonella	0.04	0.426		0.205	
Bilophila	-0.07	0.173		0.118	
Parasutterella	-0.03	0.538		0.225	
Sutterella	-0.04	0.463		0.205	
Escherichia-Shigella	0.06	0.248		0.150	

UCG.004, Lachnospiraceae_UCG-004, ***p* or *q* <.01, **p* or *q* <.05, †*q* <.10.

Supplementary Table 2. Statistical Comparisons of Intestinal Microbiomes between the High- and Low-BDI Groups Using the Mann–Whitney U test

		All participants (N = 344)		Low-BDI Group (N = 296)		High-BDI Group (N = 48)		Mann-Whitney *U* test (Low vs High BDI Group)		
		Mean	*SD*	*Mean*	*SD*	*Mean*	*SD*	*W*	*p* -value	*q* -value
Intestinal microbiome	Shannonα	6.12	0.48	6.14	0.47	6.02	0.51	8027.00	0.149	0.236
	Actinomyces	-4.02	0.82	-4.03	0.82	-3.94	0.86	6704.00	0.519	0.370
	Bifidobacterium	-1.24	0.38	-1.22	0.30	-1.34	0.69	7197.00	0.884	0.431
	Collinsella	-2.76	1.60	-2.77	1.60	-2.69	1.61	6738.00	0.560	0.370
	Eggerthella	-2.94	0.78	-2.95	0.77	-2.91	0.80	6748.00	0.577	0.371
	Bacteroides	-0.56	0.15	-0.56	0.14	-0.57	0.20	6913.00	0.765	0.431
	Odoribacter	-3.13	1.16	-3.13	1.16	-3.17	1.17	7488.00	0.544	0.370
	Alistipes	-2.21	1.20	-2.19	1.18	-2.35	1.27	7644.00	0.398	0.370
	Parabacteroides	-1.88	1.09	-1.89	1.08	-1.81	1.14	6051.50	0.099	0.178
	Erysipelatoclostridium	-2.80	0.79	-2.81	0.81	-2.72	0.67	6930.00	0.785	0.431
	Turicibacter	-3.91	1.04	-3.89	1.04	-4.02	1.08	7562.00	0.455	0.370
	Streptococcus	-2.09	0.69	-2.08	0.66	-2.14	0.86	6957.00	0.818	0.431
	Intestinibacter	-3.43	1.04	-3.43	1.04	-3.45	1.08	7095.00	0.989	0.461
	Romboutsia	-3.71	1.13	-3.69	1.12	-3.85	1.21	7525.00	0.495	0.370
	Agathobacter	-3.04	1.54	-3.01	1.54	-3.19	1.56	7597.00	0.429	0.370
	Anaerostipes	-2.07	0.74	-2.04	0.70	-2.27	0.93	8019.00	0.152	0.236
	Blautia	-1.31	0.23	-1.32	0.23	-1.28	0.23	6404.00	0.273	0.312
	Dorea	-2.40	1.05	-2.38	1.04	-2.55	1.16	7658.00	0.386	0.370
	Fusicatenibacter	-2.45	1.30	-2.43	1.28	-2.62	1.43	7375.00	0.670	0.393
	Lachnoclostridium	-1.86	0.36	-1.88	0.36	-1.74	0.34	5536.00	0.014 *	0.037 *
	Lachnospira	-2.70	1.17	-2.63	1.12	-3.13	1.34	8672.50	0.014 *	0.037 *
	Roseburia	-3.20	1.23	-3.16	1.20	-3.43	1.40	7789.50	0.279	0.312
	Sellimonas	-2.99	1.11	-2.98	1.12	-3.06	1.11	7500.00	0.534	0.370
	Tuzzerella	-3.89	1.05	-3.86	1.05	-4.06	1.08	7721.00	0.312	0.312
	UCG.004	-3.59	1.20	-3.59	1.20	-3.58	1.25	7061.00	0.945	0.447
	Colidextribacter	-3.04	1.05	-3.04	1.06	-3.01	1.04	6972.50	0.836	0.431
	Flavonifractor	-2.96	0.75	-2.96	0.74	-2.94	0.81	6700.00	0.527	0.370
	Oscillibacter	-2.88	0.91	-2.88	0.91	-2.90	0.95	7207.00	0.872	0.431
	Butyricicoccus	-2.60	0.61	-2.59	0.60	-2.66	0.63	7286.00	0.776	0.431
	Faecalibacterium	-1.53	0.97	-1.47	0.88	-1.89	1.37	8442.50	0.036 *	0.077 †
	Negativibacillus	-3.94	1.04	-3.93	1.05	-4.02	1.02	7482.50	0.533	0.370
	Subdoligranulum	-3.07	1.37	-3.02	1.34	-3.36	1.47	7928.50	0.192	0.284
	UBA1819	-3.72	0.94	-3.71	0.92	-3.81	1.06	7422.50	0.613	0.379
	Monoglobus	-2.91	1.15	-2.88	1.14	-3.12	1.20	7846.00	0.244	0.312
	Phascolarctobacterium	-3.19	1.48	-3.17	1.46	-3.34	1.59	7179.00	0.904	0.433
	Veillonella	-3.25	1.51	-3.26	1.51	-3.16	1.58	6808.00	0.634	0.379
	Bilophila	-3.11	1.17	-3.10	1.16	-3.17	1.24	7014.00	0.887	0.431
	Parasutterella	-3.18	1.47	-3.14	1.46	-3.40	1.52	7750.00	0.301	0.312
	Sutterella	-3.03	1.64	-3.00	1.63	-3.21	1.69	7398.00	0.635	0.379
	Escherichia-Shigella	-3.52	1.33	-3.57	1.32	-3.26	1.39	6137.50	0.117	0.200

UCG.004, Lachnospiraceae_UCG-004, ***p* or *q* <.01, **p* or *q* <.05, †*q* <.10.

Supplementary Table 3. Statistical Comparisons of the Intestinal Microbiomes between the High- and Low-BDI Groups Using ANCOVA with Covariates

**Shannon α**	Sum Sq	df	*F*	*p*	
Group (BDI high vs low)	13394.00	1.00	1.40	0.24	
Education	36984.00	1.00	3.86	0.05	
Number of children	20690.00	1.00	2.16	0.14	
Staple food	50303.00	1.00	5.25	0.02	*
Fruits	134.00	1.00	0.01	0.91	
Yogurt	4274.00	1.00	0.45	0.50	
Milk and cheese	30343.00	1.00	3.17	0.08	
Meat	21847.00	1.00	2.28	0.13	
Pickled vegetables	1490.00	1.00	0.16	0.69	
Residuals	3074267.00	321.00			

**Actinomyces**	Sum Sq	df	*F*-value	*p*	
Group (BDI high vs low)	521.00	1.00	0.06	0.81	
Education	469.00	1.00	0.05	0.82	
Number of children	18560.00	1.00	1.96	0.16	
Staple food	3390.00	1.00	0.36	0.55	
Fruits	1996.00	1.00	0.21	0.65	
Yogurt	12.00	1.00	0.00	0.97	
Milk and cheese	797.00	1.00	0.08	0.77	
Meat	1829.00	1.00	0.19	0.66	
Pickled vegetables	8802.00	1.00	0.93	0.34	
Residuals	3034771.00	321.00			

**Bifidobacterium**	Sum Sq	df	*F*-value	*p*	
Group (BDI high vs low)	304.00	1.00	0.03	0.86	
Education	2893.00	1.00	0.30	0.58	
Number of children	655.00	1.00	0.07	0.79	
Staple food	415.00	1.00	0.04	0.83	
Fruits	290.00	1.00	0.03	0.86	
Yogurt	13.00	1.00	0.00	0.97	
Milk and cheese	122174.00	1.00	12.84	0.00	***
Meat	15023.00	1.00	1.58	0.21	
Pickled vegetables	37470.00	1.00	3.94	0.05	*
Residuals	3053972.00	321.00			

**Collinsella**	Sum Sq	df	*F*-value	*p*	
Group (BDI high vs low)	1727.00	1.00	0.19	0.67	
Education	50.00	1.00	0.01	0.94	
Number of children	7897.00	1.00	0.85	0.36	
Staple food	4237.00	1.00	0.46	0.50	
Fruits	58320.00	1.00	6.29	0.01	*
Yogurt	2623.00	1.00	0.28	0.60	
Milk and cheese	751.00	1.00	0.08	0.78	
Meat	3766.00	1.00	0.41	0.52	
Pickled vegetables	31009.00	1.00	3.35	0.07	
Residuals	2973993.00	321.00			

**Eggerthella**	Sum Sq	df	*F*-value	*p*	
Group (BDI high vs low)	6828.00	1.00	0.70	0.40	
Education	2135.00	1.00	0.22	0.64	
Number of children	894.00	1.00	0.09	0.76	
Staple food	124502.00	1.00	12.71	0.00	***
Fruits	1896.00	1.00	0.19	0.66	
Yogurt	9870.00	1.00	1.01	0.32	
Milk and cheese	332.00	1.00	0.03	0.85	
Meat	3994.00	1.00	0.41	0.52	
Pickled vegetables	2752.00	1.00	0.28	0.60	
Residuals	3144404.00	321.00			

**Turicibacter**	Sum Sq	df	*F*-value	*p*	
Group (BDI high vs low)	16736.00	1.00	1.88	0.17	
Education	24360.00	1.00	2.74	0.10	
Number of children	1305.00	1.00	0.15	0.70	
Staple food	15459.00	1.00	1.74	0.19	
Fruits	13729.00	1.00	1.55	0.21	
Yogurt	2198.00	1.00	0.25	0.62	
Milk and cheese	9446.00	1.00	1.06	0.30	
Meat	49759.00	1.00	5.60	0.02	*
Pickled vegetables	4707.00	1.00	0.53	0.47	
Residuals	2850198.00	321.00			

**Streptococcus**	Sum Sq	df	*F*-value	*p*	
Group (BDI high vs low)	801.00	1.00	0.08	0.77	
Education	42315.00	1.00	4.46	0.04	*
Number of children	18938.00	1.00	1.99	0.16	
Staple food	28426.00	1.00	2.99	0.08	
Fruits	17509.00	1.00	1.84	0.18	
Yogurt	29135.00	1.00	3.07	0.08	
Milk and cheese	11099.00	1.00	1.17	0.28	
Meat	3472.00	1.00	0.37	0.55	
Pickled vegetables	7758.00	1.00	0.82	0.37	
Residuals	3048670.00	321.00			

**Intestinibacter**	Sum Sq	df	*F*-value	*p*	
Group (BDI high vs low)	689.00	1.00	0.07	0.79	
Education	30108.00	1.00	3.11	0.08	
Number of children	12536.00	1.00	1.29	0.26	
Staple food	9470.00	1.00	0.98	0.32	
Fruits	4480.00	1.00	0.46	0.50	
Yogurt	16002.00	1.00	1.65	0.20	
Milk and cheese	1497.00	1.00	0.15	0.69	
Meat	28208.00	1.00	2.91	0.09	
Pickled vegetables	1032.00	1.00	0.11	0.74	
Residuals	3110898.00	321.00			

**Romboutsia**	Sum Sq	df	*F*-value	*p*	
Group (BDI high vs low)	12393.00	1.00	1.32	0.25	
Education	16889.00	1.00	1.80	0.18	
Number of children	44.00	1.00	0.00	0.95	
Staple food	8505.00	1.00	0.90	0.34	
Fruits	1364.00	1.00	0.15	0.70	
Yogurt	21811.00	1.00	2.32	0.13	
Milk and cheese	982.00	1.00	0.10	0.75	
Meat	293.00	1.00	0.03	0.86	
Pickled vegetables	6822.00	1.00	0.73	0.39	
Residuals	3018148.00	321.00			

**Agathobacter**	Sum Sq	df	*F*-value	*p*	
Group (BDI high vs low)	1044.00	1.00	0.11	0.74	
Education	2740.00	1.00	0.30	0.59	
Number of children	52104.00	1.00	5.65	0.02	*
Staple food	522.00	1.00	0.06	0.81	
Fruits	7109.00	1.00	0.77	0.38	
Yogurt	8784.00	1.00	0.95	0.33	
Milk and cheese	34129.00	1.00	3.70	0.06	
Meat	15893.00	1.00	1.72	0.19	
Pickled vegetables	5218.00	1.00	0.57	0.45	
Residuals	2958409.00	321.00			

Bacteroides	Sum Sq	df	*F*-value	*p*	
Group (BDI high vs low)	273.00	1.00	0.03	0.87	
Education	9882.00	1.00	1.03	0.31	
Number of children	9828.00	1.00	1.02	0.31	
Staple food	117718.00	1.00	12.23	0.00	***
Fruits	11359.00	1.00	1.18	0.28	
Yogurt	1205.00	1.00	0.13	0.72	
Milk and cheese	1008.00	1.00	0.10	0.75	
Meat	415.00	1.00	0.04	0.84	
Pickled vegetables	376.00	1.00	0.04	0.84	
Residuals	3090476.00	321.00			

Anaerostipes	Sum Sq	df	*F*-value	*p*	
Group (BDI high vs low)	24605.00	1.00	2.50	0.11	
Education	17150.00	1.00	1.74	0.19	
Number of children	8627.00	1.00	0.88	0.35	
Staple food	421.00	1.00	0.04	0.84	
Fruits	1361.00	1.00	0.14	0.71	
Yogurt	4993.00	1.00	0.51	0.48	
Milk and cheese	72.00	1.00	0.01	0.93	
Meat	3225.00	1.00	0.33	0.57	
Pickled vegetables	17305.00	1.00	1.76	0.19	
Residuals	3160996.00	321.00			

Odoribacter	Sum Sq	df	*F*-value	*p*	
Group (BDI high vs low)	1648.00	1.00	0.17	0.68	
Education	315.00	1.00	0.03	0.86	
Number of children	2140.00	1.00	0.22	0.64	
Staple food	2510.00	1.00	0.25	0.62	
Fruits	96.00	1.00	0.01	0.92	
Yogurt	405.00	1.00	0.04	0.84	
Milk and cheese	3836.00	1.00	0.39	0.53	
Meat	80.00	1.00	0.01	0.93	
Pickled vegetables	519.00	1.00	0.05	0.82	
Residuals	3184073.00	321.00			

Blautia	Sum Sq	df	*F*-value	*p*	
Group (BDI high vs low)	7413.00	1.00	0.74	0.39	
Education	141.00	1.00	0.01	0.91	
Number of children	208.00	1.00	0.02	0.89	
Staple food	17903.00	1.00	1.78	0.18	
Fruits	10441.00	1.00	1.04	0.31	
Yogurt	1982.00	1.00	0.20	0.66	
Milk and cheese	736.00	1.00	0.07	0.79	
Meat	5631.00	1.00	0.56	0.45	
Pickled vegetables	3983.00	1.00	0.40	0.53	
Residuals	3227777.00	321.00			

Alistipes	Sum Sq	df	*F*-value	*p*	
Group (BDI high vs low)	6857.00	1.00	0.70	0.40	
Education	1720.00	1.00	0.18	0.68	
Number of children	11244.00	1.00	1.15	0.29	
Staple food	22490.00	1.00	2.29	0.13	
Fruits	9201.00	1.00	0.94	0.33	
Yogurt	286.00	1.00	0.03	0.86	
Milk and cheese	10729.00	1.00	1.09	0.30	
Meat	27945.00	1.00	2.85	0.09	
Pickled vegetables	8963.00	1.00	0.91	0.34	
Residuals	3148150.00	321.00			

Dorea	Sum Sq	df	*F*-value	*p*	
Group (BDI high vs low)	6338.00	1.00	0.63	0.43	
Education	2084.00	1.00	0.21	0.65	
Number of children	11.00	1.00	0.00	0.97	
Staple food	304.00	1.00	0.03	0.86	
Fruits	40875.00	1.00	4.09	0.04	*
Yogurt	220.00	1.00	0.02	0.88	
Milk and cheese	5290.00	1.00	0.53	0.47	
Meat	49878.00	1.00	4.98	0.03	*
Pickled vegetables	217.00	1.00	0.02	0.88	
Residuals	3211917.00	321.00			

Parabacteroides	Sum Sq	df	*F*-value	*p*	
Group (BDI high vs low)	31770.00	1.00	3.21	0.07	
Education	6679.00	1.00	0.67	0.41	
Number of children	22151.00	1.00	2.24	0.14	
Staple food	1448.00	1.00	0.15	0.70	
Fruits	17905.00	1.00	1.81	0.18	
Yogurt	61980.00	1.00	6.26	0.01	*
Milk and cheese	109.00	1.00	0.01	0.92	
Meat	5040.00	1.00	0.51	0.48	
Pickled vegetables	5039.00	1.00	0.51	0.48	
Residuals	3178334.00	321.00			

Fusicatenibacter	Sum Sq	df	*F*-value	*p*	
Group (BDI high vs low)	351.00	1.00	0.04	0.85	
Education	974.00	1.00	0.10	0.75	
Number of children	5119.00	1.00	0.51	0.47	
Staple food	6176.00	1.00	0.62	0.43	
Fruits	645.00	1.00	0.06	0.80	
Yogurt	1179.00	1.00	0.12	0.73	
Milk and cheese	0.00	1.00	0.00	1.00	
Meat	17.00	1.00	0.00	0.97	
Pickled vegetables	15767.00	1.00	1.58	0.21	
Residuals	3193619.00	321.00			

Erysipelatoclostridium	Sum Sq	df	*F*-value	*p*	
Group (BDI high vs low)	101.00	1.00	0.01	0.92	
Education	3645.00	1.00	0.37	0.54	
Number of children	14731.00	1.00	1.51	0.22	
Staple food	59621.00	1.00	6.10	0.01	*
Fruits	2354.00	1.00	0.24	0.62	
Yogurt	27010.00	1.00	2.77	0.10	
Milk and cheese	1719.00	1.00	0.18	0.68	
Meat	7467.00	1.00	0.76	0.38	
Pickled vegetables	24.00	1.00	0.00	0.96	
Residuals	3135518.00	321.00			

Lachnoclostridium	Sum Sq	df	*F*-value	*p*	
Group (BDI high vs low)	33571.00	1.00	3.45	0.06	
Education	8699.00	1.00	0.89	0.35	
Number of children	7543.00	1.00	0.78	0.38	
Staple food	49091.00	1.00	5.05	0.03	*
Fruits	13509.00	1.00	1.39	0.24	
Yogurt	8612.00	1.00	0.89	0.35	
Milk and cheese	3713.00	1.00	0.38	0.54	
Meat	4790.00	1.00	0.49	0.48	
Pickled vegetables	579.00	1.00	0.06	0.81	
Residuals	3122707.00	321.00			

Lachnospira	Sum Sq	df	*F*-value	*p*	
Group (BDI high vs low)	39856.00	1.00	4.25	0.04	*
Education	9420.00	1.00	1.00	0.32	
Number of children	61.00	1.00	0.01	0.94	
Staple food	1876.00	1.00	0.20	0.66	
Fruits	21387.00	1.00	2.28	0.13	
Yogurt	1160.00	1.00	0.12	0.73	
Milk and cheese	7526.00	1.00	0.80	0.37	
Meat	934.00	1.00	0.10	0.75	
Pickled vegetables	63871.00	1.00	6.81	0.01	**
Residuals	3011348.00	321.00			

Roseburia	Sum Sq	df	*F*-value	*p*	
Group (BDI high vs low)	19064.00	1.00	1.99	0.16	
Education	5532.00	1.00	0.58	0.45	
Number of children	202.00	1.00	0.02	0.88	
Staple food	5921.00	1.00	0.62	0.43	
Fruits	102.00	1.00	0.01	0.92	
Yogurt	1180.00	1.00	0.12	0.73	
Milk and cheese	30514.00	1.00	3.18	0.08	
Meat	8144.00	1.00	0.85	0.36	
Pickled vegetables	37973.00	1.00	3.95	0.05	*
Residuals	3082915.00	321.00			

Sellimonas	Sum Sq	df	*F*-value	*p*	
Group (BDI high vs low)	827.00	1.00	0.08	0.77	
Education	381.00	1.00	0.04	0.84	
Number of children	17766.00	1.00	1.81	0.18	
Staple food	39507.00	1.00	4.02	0.05	*
Fruits	25326.00	1.00	2.58	0.11	
Yogurt	12625.00	1.00	1.29	0.26	
Milk and cheese	780.00	1.00	0.08	0.78	
Meat	903.00	1.00	0.09	0.76	
Pickled vegetables	9957.00	1.00	1.01	0.31	
Residuals	3153117.00	321.00			

Tuzzerella	Sum Sq	df	*F*-value	*p*
Group (BDI high vs low)	6755.00	1.00	0.76	0.38
Education	24124.00	1.00	2.73	0.10
Number of children	5898.00	1.00	0.67	0.41
Staple food	510.00	1.00	0.06	0.81
Fruits	2317.00	1.00	0.26	0.61
Yogurt	19134.00	1.00	2.16	0.14
Milk and cheese	13889.00	1.00	1.57	0.21
Meat	17721.00	1.00	2.00	0.16
Pickled vegetables	1374.00	1.00	0.16	0.69
Residuals	2838626.00	321.00		

Lachnospiraceae_UCG-004	Sum Sq	df	*F*-value	*p*
Group (BDI high vs low)	4019.00	1.00	0.43	0.51
Education	63.00	1.00	0.01	0.93
Number of children	16698.00	1.00	1.80	0.18
Staple food	12410.00	1.00	1.34	0.25
Fruits	13117.00	1.00	1.42	0.23
Yogurt	5464.00	1.00	0.59	0.44
Milk and cheese	239.00	1.00	0.03	0.87
Meat	7045.00	1.00	0.76	0.38
Pickled vegetables	5500.00	1.00	0.59	0.44
Residuals	2972213.00	321.00		

Negativibacillus	Sum Sq	df	*F*-value	*p*
Group (BDI high vs low)	457.00	1.00	0.05	0.82
Education	11097.00	1.00	1.29	0.26
Number of children	30236.00	1.00	3.51	0.06
Staple food	23227.00	1.00	2.70	0.10
Fruits	20921.00	1.00	2.43	0.12
Yogurt	18478.00	1.00	2.14	0.14
Milk and cheese	7009.00	1.00	0.81	0.37
Meat	22947.00	1.00	2.66	0.10
Pickled vegetables	31060.00	1.00	3.60	0.06
Residuals	2766293.00	321.00		

Subdoligranulum	Sum Sq	df	*F*-value	*p*
Group (BDI high vs low)	9047.00	1.00	0.92	0.34
Education	104.00	1.00	0.01	0.92
Number of children	1016.00	1.00	0.10	0.75
Staple food	2506.00	1.00	0.26	0.61
Fruits	267.00	1.00	0.03	0.87
Yogurt	847.00	1.00	0.09	0.77
Milk and cheese	9.00	1.00	0.00	0.98
Meat	17126.00	1.00	1.74	0.19
Pickled vegetables	8769.00	1.00	0.89	0.35
Residuals	3150840.00	321.00		

UBA1819	Sum Sq	df	*F*-value	*p*
Group (BDI high vs low)	1612.00	1.00	0.17	0.68
Education	27824.00	1.00	2.87	0.09
Number of children	351.00	1.00	0.04	0.85
Staple food	18796.00	1.00	1.94	0.16
Fruits	30.00	1.00	0.00	0.96
Yogurt	6.00	1.00	0.00	0.98
Milk and cheese	22716.00	1.00	2.34	0.13
Meat	1.00	1.00	0.00	0.99
Pickled vegetables	16643.00	1.00	1.72	0.19
Residuals	3112511.00	321.00		

Monoglobus	Sum Sq	df	*F*-value	*p*
Group (BDI high vs low)	3288.00	1.00	0.34	0.56
Education	3218.00	1.00	0.33	0.57
Number of children	10231.00	1.00	1.05	0.31
Staple food	2768.00	1.00	0.28	0.59
Fruits	10341.00	1.00	1.06	0.30
Yogurt	19194.00	1.00	1.97	0.16
Milk and cheese	618.00	1.00	0.06	0.80
Meat	7431.00	1.00	0.76	0.38
Pickled vegetables	23099.00	1.00	2.37	0.12
Residuals	3128796.00	321.00		

Phascolarctobacterium	Sum Sq	df	*F*-value	*p*
Group (BDI high vs low)	1562.00	1.00	0.17	0.68
Education	1165.00	1.00	0.13	0.72
Number of children	6242.00	1.00	0.67	0.41
Staple food	10660.00	1.00	1.15	0.29
Fruits	43.00	1.00	0.00	0.95
Yogurt	3644.00	1.00	0.39	0.53
Milk and cheese	13580.00	1.00	1.46	0.23
Meat	630.00	1.00	0.07	0.79
Pickled vegetables	65.00	1.00	0.01	0.93
Residuals	2986113.00	321.00		

Colidextribacter	Sum Sq	df	*F*-value	*p*	
Group (BDI high vs low)	48.00	1.00	0.00	0.94	
Education	7459.00	1.00	0.76	0.38	
Number of children	1402.00	1.00	0.14	0.71	
Staple food	132.00	1.00	0.01	0.91	
Fruits	13031.00	1.00	1.33	0.25	
Yogurt	296.00	1.00	0.03	0.86	
Milk and cheese	15889.00	1.00	1.62	0.20	
Meat	35053.00	1.00	3.57	0.06	
Pickled vegetables	40417.00	1.00	4.11	0.04	*
Residuals	3154925.00	321.00			

Veillonella	Sum Sq	df	*F*-value	*p*	
Group (BDI high vs low)	2447.00	1.00	0.27	0.61	
Education	5564.00	1.00	0.60	0.44	
Number of children	8049.00	1.00	0.87	0.35	
Staple food	101711.00	1.00	11.05	0.00	***
Fruits	19.00	1.00	0.00	0.96	
Yogurt	20998.00	1.00	2.28	0.13	
Milk and cheese	26751.00	1.00	2.91	0.09	
Meat	11549.00	1.00	1.25	0.26	
Pickled vegetables	5895.00	1.00	0.64	0.42	
Residuals	2955231.00	321.00			

Flavonifractor	Sum Sq	df	*F*-value	*p*	
Group (BDI high vs low)	7585.00	1.00	0.76	0.38	
Education	1079.00	1.00	0.11	0.74	
Number of children	265.00	1.00	0.03	0.87	
Staple food	50712.00	1.00	5.09	0.02	*
Fruits	3121.00	1.00	0.31	0.58	
Yogurt	46493.00	1.00	4.67	0.03	*
Milk and cheese	7907.00	1.00	0.79	0.37	
Meat	150.00	1.00	0.02	0.90	
Pickled vegetables	12393.00	1.00	1.24	0.27	
Residuals	3198743.00	321.00			

Bilophila	Sum Sq	df	*F*-value	*p*	
Group (BDI high vs low)	3091.00	1.00	0.32	0.57	
Education	633.00	1.00	0.06	0.80	
Number of children	1443.00	1.00	0.15	0.70	
Staple food	6774.00	1.00	0.69	0.41	
Fruits	17262.00	1.00	1.77	0.18	
Yogurt	32346.00	1.00	3.31	0.07	
Milk and cheese	3393.00	1.00	0.35	0.56	
Meat	19053.00	1.00	1.95	0.16	
Pickled vegetables	2267.00	1.00	0.23	0.63	
Residuals	3132620.00	321.00			

Oscillibacter	Sum Sq	df	*F*-value	*p*	
Group (BDI high vs low)	337	1	0.03	0.85	
Education	40677	1	4.13	0.04	*
Number of children	279	1	0.03	0.87	
Staple food	6125	1	0.62	0.43	
Fruits	2402	1	0.24	0.62	
Yogurt	3048	1	0.31	0.58	
Milk and cheese	19340	1	1.96	0.16	
Meat	10482	1	1.06	0.30	
Pickled vegetables	8391	1	0.85	0.36	
Residuals	3161750	321			

Parasutterella	Sum Sq	df	*F*-value	*p*	
Group (BDI high vs low)	2924.00	1.00	0.31	0.58	
Education	4141.00	1.00	0.45	0.50	
Number of children	49331.00	1.00	5.31	0.02	*
Staple food	9745.00	1.00	1.05	0.31	
Fruits	6109.00	1.00	0.66	0.42	
Yogurt	8.00	1.00	0.00	0.98	
Milk and cheese	1043.00	1.00	0.11	0.74	
Meat	782.00	1.00	0.08	0.77	
Pickled vegetables	8527.00	1.00	0.92	0.34	
Residuals	2983442.00	321.00			

Butyricicoccus	Sum Sq	df	*F*-value	*p*	
Group (BDI high vs low)	706.00	1.00	0.07	0.79	
Education	986.00	1.00	0.10	0.75	
Number of children	9786.00	1.00	0.99	0.32	
Staple food	690.00	1.00	0.07	0.79	
Fruits	147.00	1.00	0.01	0.90	
Yogurt	28.00	1.00	0.00	0.96	
Milk and cheese	40779.00	1.00	4.12	0.04	*
Meat	17305.00	1.00	1.75	0.19	
Pickled vegetables	5692.00	1.00	0.58	0.45	
Residuals	3175194.00	321.00			

Sutterella	Sum Sq	df	*F*-value	*p*	
Group (BDI high vs low)	85.00	1.00	0.01	0.92	
Education	826.00	1.00	0.09	0.77	
Number of children	2570.00	1.00	0.27	0.60	
Staple food	102.00	1.00	0.01	0.92	
Fruits	14347.00	1.00	1.53	0.22	
Yogurt	57.00	1.00	0.01	0.94	
Milk and cheese	7098.00	1.00	0.76	0.38	
Meat	9288.00	1.00	0.99	0.32	
Pickled vegetables	3219.00	1.00	0.34	0.56	
Residuals	3009778.00	321.00			

Faecalibacterium	Sum Sq	df	*F*-value	*p*	
Group (BDI high vs low)	20676.00	1.00	2.12	0.15	
Education	3427.00	1.00	0.35	0.55	
Number of children	3255.00	1.00	0.33	0.56	
Staple food	336.00	1.00	0.03	0.85	
Fruits	1884.00	1.00	0.19	0.66	
Yogurt	435.00	1.00	0.04	0.83	
Milk and cheese	958.00	1.00	0.10	0.75	
Meat	9821.00	1.00	1.01	0.32	
Pickled vegetables	6922.00	1.00	0.71	0.40	
Residuals	3132827.00	321.00			

Escherichia-Shigella	Sum Sq	df	*F*-value	*p*	
Group (BDI high vs low)	6986.00	1.00	0.77	0.38	
Education	6115.00	1.00	0.68	0.41	
Number of children	2007.00	1.00	0.22	0.64	
Staple food	39002.00	1.00	4.32	0.04	*
Fruits	24126.00	1.00	2.67	0.10	
Yogurt	1805.00	1.00	0.20	0.66	
Milk and cheese	48994.00	1.00	5.42	0.02	*
Meat	5733.00	1.00	0.63	0.43	
Pickled vegetables	1764.00	1.00	0.20	0.66	
Residuals	2901313.00	321.00			

UCG.004, Lachnospiraceae_UCG-004, *** p <.001, ** p <.01, * p <.05

Supplementary Table 4. Statistical Comparisons of Depression, Physical Condition, and Intestinal Microbiome between the High- and Low-VMD Groups Using a T-test

		Vegetable and meat dietary pattern (VMD)						Soy and fermented food dietary pattern (SFD)					
		Low group (N = 172)		High group (N = 172)		*T*-test		Low group (N = 172)		High group (N = 172)		*T*-test	
		Mean	*SD*	*Mean*	*SD*	*p*-value	*q*-value	*Mean*	*SD*	*Mean*	*SD*	*p*-value	*q*-value
Psychological and physical indices	VMD score	12.33	1.64	17.60	1.83	0.000 **	0.000 **	–	–	–	–	–	–
	SFD score	–	–	–	–	–	–	7.73	1.08	11.51	1.61	0.000 **	0.000 **
	BDI	11.70	8.58	10.32	7.68	0.119	0.180	12.05	8.70	9.98	7.47	0.019 *	0.035 *
	Mother's age	34.35	5.26	34.92	4.24	0.271	0.318	33.96	5.03	35.31	4.43	0.009 **	0.018 **
	Level of education	1.32	0.52	1.41	0.53	0.122	0.180	1.31	0.51	1.41	0.53	0.079	0.117
	Household income	1.76	1.02	1.88	1.00	0.295	0.334	1.75	1.01	1.88	1.01	0.255	0.319
	Number of children	2.04	0.88	1.99	0.79	0.597	0.597	2.05	0.92	1.99	0.75	0.511	0.572
	Age of youngest child	1.32	0.65	1.20	0.70	0.109	0.176	1.27	0.66	1.24	0.69	0.681	0.702
	Employment status	1.68	0.69	1.47	0.81	0.010 *	0.023 *	1.63	0.72	1.52	0.80	0.191	0.271
	MDPS total score	15.38	5.93	13.95	5.36	0.020 *	0.044 *	15.26	5.61	14.07	5.72	0.053	0.083 †
	Physical activity index (MDPS_PAI)	3.27	1.63	2.90	1.39	0.024 *	0.047 *	3.24	1.56	2.92	1.47	0.048 *	0.081 †
	Somatic disorders index (MDPS_SDI)	1.07	1.33	0.88	1.11	0.159	0.216	1.02	1.24	0.94	1.21	0.538	0.572
	Hormone activity index (MDPS_HAI)	4.38	1.70	4.11	1.78	0.146	0.206	4.33	1.72	4.16	1.76	0.370	0.433
	Microvascular disorders index (MDPS_MDI)	3.77	2.07	3.50	1.91	0.214	0.270	3.77	2.04	3.50	1.95	0.214	0.292
	Meteoropathy related index (MDPS_MRI)	2.89	1.71	2.56	1.58	0.063	0.112	2.90	1.65	2.55	1.63	0.054	0.083 †
	Sleeping time	6.81	1.11	6.72	1.07	0.480	0.510	6.75	1.11	6.78	1.07	0.776	0.776
	Sleeping quality	1.93	0.62	1.89	0.58	0.528	0.544	1.89	0.61	1.93	0.59	0.528	0.572
Dietary intake	Staple food	4.18	1.10	4.83	0.52	0.000 **	0.000 **	4.45	0.94	4.56	0.89	0.240	0.314
	Unrefined grain	1.34	0.85	1.47	1.03	0.230	0.280	1.13	0.56	1.68	1.15	0.000 **	0.000 **
	Root vegetable	2.57	0.77	4.02	0.75	0.000 **	0.000 **	2.88	0.98	3.70	0.95	0.000 **	0.000 **
	Green and yellow vegetables	2.31	0.59	3.84	0.82	0.000 **	0.000 **	2.64	0.92	3.51	0.99	0.000 **	0.000 **
	Light colored vegetables	2.53	0.72	3.91	0.80	0.000 **	0.000 **	2.85	0.92	3.58	1.00	0.000 **	0.000 **
	Fruits	2.19	0.93	3.09	1.00	0.000 **	0.000 **	2.13	0.87	3.15	0.99	0.000 **	0.000 **
	Meat	2.97	0.81	4.05	0.79	0.000 **	0.000 **	3.22	0.96	3.80	0.88	0.000 **	0.000 **
	Fish and shellfish	2.22	0.64	3.01	0.86	0.000 **	0.000 **	2.24	0.73	2.98	0.82	0.000 **	0.000 **
	Egg	2.71	0.84	3.33	0.80	0.000 **	0.000 **	2.73	0.82	3.30	0.85	0.000 **	0.000 **
	Milk and cheese	2.58	1.14	3.35	1.08	0.000 **	0.000 **	2.51	1.05	3.42	1.12	0.000 **	0.000 **
	Yogurt and lactic fermenting beverage	2.18	1.01	2.43	1.12	0.031 *	0.059 †	1.81	0.77	2.80	1.11	0.000 **	0.000 **
	Soy product	2.33	0.84	2.88	0.94	0.000 **	0.000 **	2.02	0.54	3.19	0.88	0.000 **	0.000 **
	Natto	1.82	0.90	1.99	0.87	0.078 *	0.132	1.56	0.67	2.24	0.95	0.000 **	0.000 **
	Pickled vegetables	1.63	0.68	1.74	0.83	0.176	0.230	1.53	0.58	1.84	0.87	0.000 **	0.000 **
	Seaweed	1.88	0.62	2.31	0.84	0.000 **	0.000 **	1.77	0.57	2.42	0.80	0.000 **	0.000 **
	Mushroom	2.26	0.66	2.90	0.92	0.000 **	0.000 **	2.12	0.63	3.03	0.83	0.000 **	0.000 **
	Snack	3.33	1.11	3.58	0.88	0.021 *	0.045 *	3.39	1.03	3.51	0.99	0.262	0.319
	Sugary drink	2.82	1.23	2.92	1.16	0.418	0.458	3.10	1.19	2.64	1.16	0.000 **	0.001 **

**p or $q < .01$, *p or $q < .05$, †$q < .10$.

Supplementary Table 5. Statistical Comparisons of Depression, Physical Condition, and Intestinal Microbiome between High- and Low-VMD Groups via ANCOVA with Covariates

**BDI**	Sum Sq	df	*F*	*p*	
Group (VMD High vs Low)	190.00	1.00	2.91	0.09	
Education	259.30	1.00	3.97	0.05	*
Number of children	180.70	1.00	2.77	0.10	
Employment status	115.60	1.00	1.77	0.18	
Residuals	20807.60	319.00			

**MDPS total**	Sum Sq	df	*F*	*p*	
Group (VMD High vs Low)	177.80	1.00	5.68	0.02	*
Education	0.30	1.00	0.01	0.93	
Number of children	118.10	1.00	3.78	0.05	
Employment status	0.50	1.00	0.01	0.90	
Residuals	10169.70	325.00			

**Sleep time**	Sum Sq	df	*F*	*p*	
Group (VMD High vs Low)	0.55	1.00	0.48	0.49	
Education	6.59	1.00	5.70	0.02	*
Number of children	8.95	1.00	7.74	0.01	**
Employment status	2.84	1.00	2.45	0.12	
Residuals	374.73	324.00			

**Sleep quality**	Sum Sq	df	*F*	*p*	
Group (VMD High vs Low)	0.00	1.00	0.01	0.93	
Education	0.08	1.00	0.23	0.63	
Number of children	0.06	1.00	0.19	0.67	
Employment status	1.81	1.00	5.22	0.02	*
Residuals	112.57	325.00			

**Shannonα**	Sum Sq	df	*F*	*p*
Group (VMD High vs Low)	1012.00	1.00	0.10	0.75
Education	30498.00	1.00	3.13	0.08
Number of children	32873.00	1.00	3.37	0.07
Employment status	13521.00	1.00	1.39	0.24
Residuals	3168024.00	325.00		

**Turicibacter**	Sum Sq	df	*F*	*p*
Group (VMD High vs Low)	492.00	1.00	0.05	0.82
Education	23012.00	1.00	2.55	0.11
Number of children	5958.00	1.00	0.66	0.42
Employment status	5019.00	1.00	0.56	0.46
Residuals	2937682.00	325.00		

**Actinomyces**	Sum Sq	df	*F*	*p*
Group (VMD High vs Low)	7319.00	1.00	0.78	0.38
Education	435.00	1.00	0.05	0.83
Number of children	22579.00	1.00	2.42	0.12
Employment status	261.00	1.00	0.03	0.87
Residuals	3032562.00	325.00		

**Streptococcus**	Sum Sq	df	*F*	*p*
Group (VMD High vs Low)	25963.00	1.00	2.67	0.10
Education	32534.00	1.00	3.35	0.07
Number of children	32493.00	1.00	3.35	0.07
Employment status	11278.00	1.00	1.16	0.28
Residuals	3156171.00	325.00		

**Bifidobacterium**	Sum Sq	df	*F*	*p*
Group (VMD High vs Low)	1364.00	1.00	0.14	0.71
Education	94.00	1.00	0.01	0.92
Number of children	1401.00	1.00	0.14	0.71
Employment status	25101.00	1.00	2.56	0.11
Residuals	3191172.00	325.00		

**Intestinibacter**	Sum Sq	df	*F*	*p*
Group (VMD High vs Low)	4384.00	1.00	0.45	0.50
Education	25434.00	1.00	2.61	0.11
Number of children	11905.00	1.00	1.22	0.27
Employment status	56.00	1.00	0.01	0.94
Residuals	3168907.00	325.00		

**Collinsella**	Sum Sq	df	*F*	*p*	
Group (VMD High vs Low)	43800.00	1.00	4.65	0.03	*
Education	979.00	1.00	0.10	0.75	
Number of children	20564.00	1.00	2.18	0.14	
Employment status	13497.00	1.00	1.43	0.23	
Residuals	3062533.00	325.00			

**Romboutsia**	Sum Sq	df	*F*	*p*
Group (VMD High vs Low)	279.00	1.00	0.03	0.86
Education	14092.00	1.00	1.49	0.22
Number of children	1207.00	1.00	0.13	0.72
Employment status	11609.00	1.00	1.23	0.27
Residuals	3066762.00	325.00		

**Eggerthella**	Sum Sq	df	*F*	*p*
Group (VMD High vs Low)	10668.00	1.00	1.07	0.30
Education	2326.00	1.00	0.23	0.63
Number of children	227.00	1.00	0.02	0.88
Employment status	33066.00	1.00	3.31	0.07
Residuals	3244872.00	325.00		

**Agathobacter**	Sum Sq	df	*F*	*p*	
Group (VMD High vs Low)	13711.00	1.00	1.48	0.22	
Education	3141.00	1.00	0.34	0.56	
Number of children	46231.00	1.00	4.99	0.03	*
Employment status	1089.00	1.00	0.12	0.73	
Residuals	3013217.00	325.00			

**Bacteroides**	Sum Sq	df	*F*	*p*
Group (VMD High vs Low)	3744.00	1.00	0.38	0.54
Education	10129.00	1.00	1.04	0.31
Number of children	13269.00	1.00	1.36	0.24
Employment status	29013.00	1.00	2.98	0.09
Residuals	3164701.00	325.00		

**Anaerostipes**	Sum Sq	df	*F*	*p*
Group (VMD High vs Low)	3486.00	1.00	0.35	0.55
Education	12284.00	1.00	1.24	0.27
Number of children	5262.00	1.00	0.53	0.47
Employment status	884.00	1.00	0.09	0.77
Residuals	3210965.00	325.00		

**Odoribacter**	Sum Sq	df	*F*	*p*
Group (VMD High vs Low)	802.00	1.00	0.08	0.77
Education	435.00	1.00	0.04	0.83
Number of children	3907.00	1.00	0.40	0.53
Employment status	10706.00	1.00	1.09	0.30
Residuals	3180319.00	325.00		

**Alistipes**	Sum Sq	df	*F*	*p*
Group (VMD High vs Low)	1506.00	1.00	0.15	0.70
Education	1019.00	1.00	0.10	0.75
Number of children	21619.00	1.00	2.18	0.14
Employment status	2808.00	1.00	0.28	0.60
Residuals	3227482.00	325.00		

**Parabacteroides**	Sum Sq	df	*F*	*p*
Group (VMD High vs Low)	2037.00	1.00	0.20	0.65
Education	7519.00	1.00	0.75	0.39
Number of children	26260.00	1.00	2.61	0.11
Employment status	11429.00	1.00	1.14	0.29
Residuals	3264407.00	325.00		

**Erysipelatoclostridium**	Sum Sq	df	*F*	*p*
Group (VMD High vs Low)	193.00	1.00	0.02	0.89
Education	2507.00	1.00	0.26	0.61
Number of children	23698.00	1.00	2.42	0.12
Employment status	34814.00	1.00	3.55	0.06
Residuals	3183507.00	325.00		

**Blautia**	Sum Sq	df	*F*	*p*
Group (VMD High vs Low)	1425.00	1.00	0.14	0.71
Education	1490.00	1.00	0.15	0.70
Number of children	356.00	1.00	0.04	0.85
Employment status	5696.00	1.00	0.57	0.45
Residuals	3249730.00	325.00		

**Dorea**	Sum Sq	df	*F*	*p*
Group (VMD High vs Low)	1164.00	1.00	0.11	0.74
Education	241.00	1.00	0.02	0.88
Number of children	346.00	1.00	0.03	0.85
Employment status	43.00	1.00	0.00	0.95
Residuals	3303489.00	325.00		

**Fusicatenibacter**	Sum Sq	df	*F*	*p*
Group (VMD High vs Low)	622.00	1.00	0.06	0.80
Education	766.00	1.00	0.08	0.78
Number of children	6675.00	1.00	0.68	0.41
Employment status	14830.00	1.00	1.51	0.22
Residuals	3185627.00	325.00		

**Lachnoclostridium**	Sum Sq	df	*F*	*p*
Group (VMD High vs Low)	12028.00	1.00	1.22	0.27
Education	20502.00	1.00	2.08	0.15
Number of children	18681.00	1.00	1.90	0.17
Employment status	25449.00	1.00	2.59	0.11
Residuals	3197504.00	325.00		

**Lachnospira**	Sum Sq	df	*F*	*p*
Group (VMD High vs Low)	11542.00	1.00	1.19	0.28
Education	4811.00	1.00	0.49	0.48
Number of children	272.00	1.00	0.03	0.87
Employment status	5840.00	1.00	0.60	0.44
Residuals	3162392.00	325.00		

**Roseburia**	Sum Sq	df	*F*	*p*
Group (VMD High vs Low)	9.00	1.00	0.00	0.98
Education	4220.00	1.00	0.44	0.51
Number of children	1210.00	1.00	0.13	0.72
Employment status	26362.00	1.00	2.72	0.10
Residuals	3145332.00	325.00		

**Sellimonas**	Sum Sq	df	*F*	*p*
Group (VMD High vs Low)	297.00	1.00	0.03	0.86
Education	1601.00	1.00	0.16	0.69
Number of children	19268.00	1.00	1.95	0.16
Employment status	14030.00	1.00	1.42	0.23
Residuals	3210994.00	325.00		

**Tuzzerella**	Sum Sq	df	*F*	*p*
Group (VMD High vs Low)	11776.00	1.00	1.33	0.25
Education	17202.00	1.00	1.94	0.16
Number of children	3192.00	1.00	0.36	0.55
Employment status	4146.00	1.00	0.47	0.49
Residuals	2877596.00	325.00		

**Negativibacillus**	Sum Sq	df	*F*	*p*	
Group (VMD High vs Low)	6580.00	1.00	0.75	0.39	
Education	12684.00	1.00	1.44	0.23	
Number of children	35796.00	1.00	4.06	0.04	*
Employment status	14.00	1.00	0.00	0.97	
Residuals	2867448.00	325.00			

**Subdoligranulum**	Sum Sq	df	*F*	*p*
Group (VMD High vs Low)	28627.00	1.00	2.95	0.09
Education	184.00	1.00	0.02	0.89
Number of children	1.00	1.00	0.00	0.99
Employment status	12129.00	1.00	1.25	0.26
Residuals	3149251.00	325.00		

**UBA1819**	Sum Sq	df	*F*	*p*	
Group (VMD High vs Low)	3860.00	1.00	0.40	0.53	
Education	24466.00	1.00	2.57	0.11	
Number of children	1616.00	1.00	0.17	0.68	
Employment status	72665.00	1.00	7.62	0.01	**
Residuals	3097984.00	325.00			

**Monoglobus**	Sum Sq	df	*F*	*p*
Group (VMD High vs Low)	13002.00	1.00	1.33	0.25
Education	2367.00	1.00	0.24	0.62
Number of children	7886.00	1.00	0.81	0.37
Employment status	28.00	1.00	0.00	0.96
Residuals	3172839.00	325.00		

Lachnospiraceae_UCG-004	Sum Sq	df	F	p	
Group (VMD High vs Low)	30763.00	1.00	3.39	0.07	
Education	616.00	1.00	0.07	0.79	
Number of children	24213.00	1.00	2.67	0.10	
Employment status	29602.00	1.00	3.26	0.07	
Residuals	2949448.00	325.00			

Phascolarctobacterium	Sum Sq	df	F	p	
Group (VMD High vs Low)	599.00	1.00	0.07	0.80	
Education	873.00	1.00	0.10	0.76	
Number of children	2236.00	1.00	0.24	0.62	
Employment status	15991.00	1.00	1.75	0.19	
Residuals	2975501.00	325.00			

Colidextribacter	Sum Sq	df	F	p	
Group (VMD High vs Low)	6551.00	1.00	0.66	0.42	
Education	5678.00	1.00	0.57	0.45	
Number of children	839.00	1.00	0.08	0.77	
Employment status	8830.00	1.00	0.88	0.35	
Residuals	3244697.00	325.00			

Veillonella	Sum Sq	df	F	p	
Group (VMD High vs Low)	1683.00	1.00	0.18	0.67	
Education	6436.00	1.00	0.68	0.41	
Number of children	4114.00	1.00	0.43	0.51	
Employment status	1925.00	1.00	0.20	0.65	
Residuals	3097203.00	325.00			

Flavonifractor	Sum Sq	df	F	p	
Group (VMD High vs Low)	643.00	1.00	0.06	0.80	
Education	1131.00	1.00	0.11	0.74	
Number of children	3772.00	1.00	0.38	0.54	
Employment status	56590.00	1.00	5.67	0.02	*
Residuals	3246030.00	325.00			

Bilophila	Sum Sq	df	F	p	
Group (VMD High vs Low)	21063.00	1.00	2.16	0.14	
Education	1524.00	1.00	0.16	0.69	
Number of children	1555.00	1.00	0.16	0.69	
Employment status	3954.00	1.00	0.41	0.52	
Residuals	3164935.00	325.00			

Oscillibacter	Sum Sq	df	F	p	
Group (VMD High vs Low)	351.00	1.00	0.04	0.85	
Education	31162.00	1.00	3.17	0.08	
Number of children	255.00	1.00	0.03	0.87	
Employment status	10807.00	1.00	1.10	0.30	
Residuals	3195230.00	325.00			

Parasutterella	Sum Sq	df	F	p	
Group (VMD High vs Low)	2144.00	1.00	0.23	0.63	
Education	8284.00	1.00	0.90	0.34	
Number of children	48165.00	1.00	5.23	0.02	*
Employment status	1942.00	1.00	0.21	0.65	
Residuals	2994534.00	325.00			

Butyricicoccus	Sum Sq	df	F	p	
Group (VMD High vs Low)	4376.00	1.00	0.44	0.51	
Education	2703.00	1.00	0.27	0.60	
Number of children	8178.00	1.00	0.83	0.36	
Employment status	11963.00	1.00	1.21	0.27	
Residuals	3210058.00	325.00			

Sutterella	Sum Sq	df	F	p	
Group (VMD High vs Low)	722.00	1.00	0.08	0.78	
Education	22.00	1.00	0.00	0.96	
Number of children	8081.00	1.00	0.89	0.35	
Employment status	74510.00	1.00	8.18	0.00	**
Residuals	2959388.00	325.00			

Faecalibacterium	Sum Sq	df	F	p	
Group (VMD High vs Low)	21191.00	1.00	2.19	0.14	
Education	870.00	1.00	0.09	0.76	
Number of children	8188.00	1.00	0.85	0.36	
Employment status	24128.00	1.00	2.49	0.12	
Residuals	3143369.00	325.00			

Escherichia-Shigella	Sum Sq	df	F	p	
Group (VMD High vs Low)	4621.00	1.00	0.50	0.48	
Education	13143.00	1.00	1.43	0.23	
Number of children	91.00	1.00	0.01	0.92	
Employment status	38178.00	1.00	4.15	0.04	*
Residuals	2991793.00	325.00			

UCG.004, Lachnospiraceae_UCG-004, ****p* <.001, ***p* <.01, **p* <.05

Supplementary Table 6. Statistical Comparisons of Depression, Physical Condition, and Intestinal Microbiome between High- and Low-SFD Groups via ANCOVA with Covariates

**BDI**	Sum Sq	df	*F*	*p*	
Group (SFD High vs Low)	302.70	1.00	4.67	0.03	*
Education	254.10	1.00	3.92	0.05	*
Number of children	185.00	1.00	2.85	0.09	
Employment status	105.60	1.00	1.63	0.20	
Residuals	20694.90	319.00			

**MDPS total**	Sum Sq	df	*F*	*p*	
Group (SFD High vs Low)	123.10	1.00	3.91	0.05	*
Education	0.40	1.00	0.01	0.91	
Number of children	117.00	1.00	3.72	0.05	
Employment status	0.00	1.00	0.00	0.97	
Residuals	10224.40	325.00			

**Sleep time**	Sum Sq	df	*F*	*p*	
Group (SFD High vs Low)	0.00	1.00	0.00	0.99	
Education	6.28	1.00	5.42	0.02	*
Number of children	8.81	1.00	7.60	0.01	**
Employment status	3.21	1.00	2.77	0.10	.
Residuals	375.28	324.00			

**Sleep quality**	Sum Sq	df	*F*	*p*	
Group (SFD High vs Low)	0.57	1.00	1.64	0.20	
Education	0.05	1.00	0.13	0.72	
Number of children	0.08	1.00	0.23	0.63	
Employment status	2.00	1.00	5.81	0.02	*
Residuals	112.01	325.00			

**Shannonα**	Sum Sq	df	*F*	*p*
Group (SFD High vs Low)	18051.00	1.00	1.86	0.17
Education	27326.00	1.00	2.82	0.09
Number of children	34161.00	1.00	3.52	0.06
Employment status	15143.00	1.00	1.56	0.21
Residuals	3150985.00	325.00		

**Turicibacter**	Sum Sq	df	*F*	*p*
Group (SFD High vs Low)	12.00	1.00	0.00	0.97
Education	23653.00	1.00	2.62	0.11
Number of children	6086.00	1.00	0.67	0.41
Employment status	5531.00	1.00	0.61	0.43
Residuals	2938162.00	325.00		

**Actinomyces**	Sum Sq	df	*F*	*p*
Group (SFD High vs Low)	225.00	1.00	0.02	0.88
Education	861.00	1.00	0.09	0.76
Number of children	23562.00	1.00	2.52	0.11
Employment status	809.00	1.00	0.09	0.77
Residuals	3039656.00	325.00		

**Streptococcus**	Sum Sq	df	*F*	*p*
Group (SFD High vs Low)	5317.00	1.00	0.54	0.46
Education	30027.00	1.00	3.07	0.08
Number of children	33465.00	1.00	3.42	0.07
Employment status	14897.00	1.00	1.52	0.22
Residuals	3176817.00	325.00		

**Bifidobacterium**	Sum Sq	df	*F*	*p*
Group (SFD High vs Low)	604.00	1.00	0.06	0.80
Education	112.00	1.00	0.01	0.92
Number of children	1374.00	1.00	0.14	0.71
Employment status	24459.00	1.00	2.49	0.12
Residuals	3191931.00	325.00		

**Intestinibacter**	Sum Sq	df	*F*	*p*
Group (SFD High vs Low)	1638.00	1.00	0.17	0.68
Education	24794.00	1.00	2.54	0.11
Number of children	12067.00	1.00	1.24	0.27
Employment status	169.00	1.00	0.02	0.90
Residuals	3171653.00	325.00		

**Collinsella**	Sum Sq	df	*F*	*p*
Group (SFD High vs Low)	28955.00	1.00	3.06	0.08
Education	1135.00	1.00	0.12	0.73
Number of children	20832.00	1.00	2.20	0.14
Employment status	17131.00	1.00	1.81	0.18
Residuals	3077378.00	325.00		

**Romboutsia**	Sum Sq	df	*F*	*p*
Group (SFD High vs Low)	13090.00	1.00	1.39	0.24
Education	12080.00	1.00	1.29	0.26
Number of children	986.00	1.00	0.10	0.75
Employment status	10353.00	1.00	1.10	0.29
Residuals	3053950.00	325.00		

**Eggerthella**	Sum Sq	df	*F*	*p*	
Group (SFD High vs Low)	5530.00	1.00	0.55	0.46	
Education	4025.00	1.00	0.40	0.53	
Number of children	436.00	1.00	0.04	0.83	
Employment status	40752.00	1.00	4.08	0.04	*
Residuals	3250010.00	325.00			

**Agathobacter**	Sum Sq	df	*F*	*p*	
Group (SFD High vs Low)	50497.00	1.00	5.51	0.02	*
Education	4377.00	1.00	0.48	0.49	
Number of children	47899.00	1.00	5.23	0.02	*
Employment status	1239.00	1.00	0.14	0.71	
Residuals	2976431.00	325.00			

**Bacteroides**	Sum Sq	df	*F*	*p*
Group (SFD High vs Low)	7762.00	1.00	0.80	0.37
Education	7686.00	1.00	0.79	0.37
Number of children	14430.00	1.00	1.48	0.22
Employment status	34560.00	1.00	3.55	0.06
Residuals	3160683.00	325.00		

**Anaerostipes**	Sum Sq	df	*F*	*p*
Group (SFD High vs Low)	5330.00	1.00	0.54	0.46
Education	12616.00	1.00	1.28	0.26
Number of children	5177.00	1.00	0.52	0.47
Employment status	775.00	1.00	0.08	0.78
Residuals	3209121.00	325.00		

Odoribacter	Sum Sq	df	*F*	*p*	
Group (SFD High vs Low)	158.00	1.00	0.02	0.90	
Education	593.00	1.00	0.06	0.81	
Number of children	4074.00	1.00	0.42	0.52	
Employment status	11806.00	1.00	1.21	0.27	
Residuals	3180962.00	325.00			

Blautia	Sum Sq	df	*F*	*p*	
Group (SFD High vs Low)	161.00	1.00	0.02	0.90	
Education	1649.00	1.00	0.16	0.69	
Number of children	327.00	1.00	0.03	0.86	
Employment status	5179.00	1.00	0.52	0.47	
Residuals	3250994.00	325.00			

Alistipes	Sum Sq	df	*F*	*p*	
Group (SFD High vs Low)	5657.00	1.00	0.57	0.45	
Education	484.00	1.00	0.05	0.83	
Number of children	22744.00	1.00	2.29	0.13	
Employment status	4102.00	1.00	0.41	0.52	
Residuals	3223331.00	325.00			

Dorea	Sum Sq	df	*F*	*p*	
Group (SFD High vs Low)	23150.00	1.00	2.29	0.13	
Education	1015.00	1.00	0.10	0.75	
Number of children	152.00	1.00	0.02	0.90	
Employment status	513.00	1.00	0.05	0.82	
Residuals	3281503.00	325.00			

Parabacteroides	Sum Sq	df	*F*	*p*	
Group (SFD High vs Low)	24509.00	1.00	2.46	0.12	
Education	9368.00	1.00	0.94	0.33	
Number of children	25023.00	1.00	2.51	0.11	
Employment status	12906.00	1.00	1.29	0.26	
Residuals	3241935.00	325.00			

Fusicatenibacter	Sum Sq	df	*F*	*p*	
Group (SFD High vs Low)	20325.00	1.00	2.09	0.15	
Education	1461.00	1.00	0.15	0.70	
Number of children	7348.00	1.00	0.75	0.39	
Employment status	16927.00	1.00	1.74	0.19	
Residuals	3165924.00	325.00			

Erysipelatoclostridium	Sum Sq	df	*F*	*p*	
Group (SFD High vs Low)	4037.00	1.00	0.41	0.52	
Education	2077.00	1.00	0.21	0.65	
Number of children	24223.00	1.00	2.48	0.12	
Employment status	36350.00	1.00	3.72	0.05	
Residuals	3179663.00	325.00			

Lachnoclostridium	Sum Sq	df	*F*	*p*	
Group (SFD High vs Low)	1040.00	1.00	0.11	0.75	
Education	17185.00	1.00	1.74	0.19	
Number of children	19957.00	1.00	2.02	0.16	
Employment status	31346.00	1.00	3.18	0.08	
Residuals	3208492.00	325.00			

Lachnospira	Sum Sq	df	*F*	*p*	
Group (SFD High vs Low)	39239.00	1.00	4.07	0.04	*
Education	6071.00	1.00	0.63	0.43	
Number of children	390.00	1.00	0.04	0.84	
Employment status	6094.00	1.00	0.63	0.43	
Residuals	3134695.00	325.00			

Negativibacillus	Sum Sq	df	*F*	*p*	
Group (SFD High vs Low)	11680.00	1.00	1.33	0.25	
Education	9256.00	1.00	1.05	0.31	
Number of children	38196.00	1.00	4.34	0.04	*
Employment status	223.00	1.00	0.03	0.87	
Residuals	2862348.00	325.00			

Roseburia	Sum Sq	df	*F*	*p*	
Group (SFD High vs Low)	3536.00	1.00	0.37	0.55	
Education	4893.00	1.00	0.51	0.48	
Number of children	1347.00	1.00	0.14	0.71	
Employment status	28020.00	1.00	2.90	0.09	
Residuals	3141806.00	325.00			

Subdoligranulum	Sum Sq	df	*F*	*p*	
Group (SFD High vs Low)	44289.00	1.00	4.59	0.03	*
Education	82.00	1.00	0.01	0.93	
Number of children	8.00	1.00	0.00	0.98	
Employment status	11007.00	1.00	1.14	0.29	
Residuals	3133588.00	325.00			

Sellimonas	Sum Sq	df	*F*	*p*	
Group (SFD High vs Low)	5359.00	1.00	0.54	0.46	
Education	2297.00	1.00	0.23	0.63	
Number of children	18434.00	1.00	1.87	0.17	
Employment status	16107.00	1.00	1.63	0.20	
Residuals	3205932.00	325.00			

UBA1819	Sum Sq	df	*F*	*p*	
Group (SFD High vs Low)	26.00	1.00	0.00	0.96	
Education	22712.00	1.00	2.38	0.12	
Number of children	1447.00	1.00	0.15	0.70	
Employment status	68962.00	1.00	7.23	0.01	**
Residuals	3101818.00	325.00			

Tuzzerella	Sum Sq	df	*F*	*p*	
Group (SFD High vs Low)	212.00	1.00	0.02	0.88	
Education	19289.00	1.00	2.17	0.14	
Number of children	3537.00	1.00	0.40	0.53	
Employment status	6032.00	1.00	0.68	0.41	
Residuals	2889160.00	325.00			

Monoglobus	Sum Sq	df	*F*	*p*	
Group (SFD High vs Low)	8766.00	1.00	0.90	0.34	
Education	2247.00	1.00	0.23	0.63	
Number of children	7970.00	1.00	0.82	0.37	
Employment status	5.00	1.00	0.00	0.98	
Residuals	3177076.00	325.00			

**Lachnospiraceae_UCG-004**	Sum Sq	df	*F*	*p*	
Group (SFD High vs Low)	1044.00	1.00	0.11	0.74	
Education	169.00	1.00	0.02	0.89	
Number of children	22825.00	1.00	2.49	0.12	
Employment status	23337.00	1.00	2.55	0.11	
Residuals	2979168.00	325.00			

**Phascolarctobacterium**	Sum Sq	df	*F*	*p*	
Group (SFD High vs Low)	12465.00	1.00	1.37	0.24	
Education	1398.00	1.00	0.15	0.70	
Number of children	1962.00	1.00	0.22	0.64	
Employment status	17557.00	1.00	1.93	0.17	
Residuals	2963635.00	325.00			

**Colidextribacter**	Sum Sq	df	*F*	*p*	
Group (SFD High vs Low)	527.00	1.00	0.05	0.82	
Education	4424.00	1.00	0.44	0.51	
Number of children	655.00	1.00	0.07	0.80	
Employment status	6766.00	1.00	0.68	0.41	
Residuals	3250722.00	325.00			

**Veillonella**	Sum Sq	df	*F*	*p*	
Group (SFD High vs Low)	2177.00	1.00	0.23	0.63	
Education	5274.00	1.00	0.55	0.46	
Number of children	3751.00	1.00	0.39	0.53	
Employment status	1242.00	1.00	0.13	0.72	
Residuals	3096709.00	325.00			

**Flavonifractor**	Sum Sq	df	*F*	*p*	
Group (SFD High vs Low)	354.00	1.00	0.04	0.85	
Education	1399.00	1.00	0.14	0.71	
Number of children	3595.00	1.00	0.36	0.55	
Employment status	54972.00	1.00	5.50	0.02	*
Residuals	3246318.00	325.00			

**Bilophila**	Sum Sq	df	*F*	*p*	
Group (SFD High vs Low)	48754.00	1.00	5.05	0.03	*
Education	2160.00	1.00	0.22	0.64	
Number of children	1340.00	1.00	0.14	0.71	
Employment status	4242.00	1.00	0.44	0.51	
Residuals	3137244.00	325.00			

**Oscillibacter**	Sum Sq	df	*F*	*p*	
Group (SFD High vs Low)	10623.00	1.00	1.08	0.30	
Education	27471.00	1.00	2.80	0.10	
Number of children	402.00	1.00	0.04	0.84	
Employment status	8878.00	1.00	0.91	0.34	
Residuals	3184957.00	325.00			

**Parasutterella**	Sum Sq	df	*F*	*p*	
Group (SFD High vs Low)	21380.00	1.00	2.34	0.13	
Education	5496.00	1.00	0.60	0.44	
Number of children	51023.00	1.00	5.57	0.02	*
Employment status	3778.00	1.00	0.41	0.52	
Residuals	2975297.00	325.00			

**Butyricicoccus**	Sum Sq	df	*F*	*p*	
Group (SFD High vs Low)	37.00	1.00	0.00	0.95	
Education	2206.00	1.00	0.22	0.64	
Number of children	8526.00	1.00	0.86	0.35	
Employment status	10380.00	1.00	1.05	0.31	
Residuals	3214397.00	325.00			

**Sutterella**	Sum Sq	df	*F*	*p*	
Group (SFD High vs Low)	417.00	1.00	0.05	0.83	
Education	18.00	1.00	0.00	0.96	
Number of children	8050.00	1.00	0.88	0.35	
Employment status	74275.00	1.00	8.16	0.00	**
Residuals	2959693.00	325.00			

**Faecalibacterium**	Sum Sq	df	*F*	*p*	
Group (SFD High vs Low)	14372.00	1.00	1.48	0.22	
Education	781.00	1.00	0.08	0.78	
Number of children	8079.00	1.00	0.83	0.36	
Employment status	21462.00	1.00	2.21	0.14	
Residuals	3150187.00	325.00			

**Escherichia-Shigella**	Sum Sq	df	*F*	*p*	
Group (SFD High vs Low)	37018.00	1.00	4.07	0.04	*
Education	10653.00	1.00	1.17	0.28	
Number of children	193.00	1.00	0.02	0.88	
Employment status	40948.00	1.00	4.50	0.03	*
Residuals	2959396.00	325.00			

UCG.004, Lachnospiraceae_UCG-004, ***p <.001, **p <.01, *p <.05

Supplementary Table 7. The list of questionnaires and orders

As part of the Japanese research project The Principle of Human Social Brain-Mind Development, this study was analyzed the following questionnaires data.

The list of questionnaires and orders.

1	The Parenting Stress Index (PSI)	**NOT** used in this study
2	The Beck Depression Inventory-II (BDI-II)	**USED** in this study
3	The Multidimensional Physical Scale (MDPS)	**USED** in this study
4	Multidimensional Assessment of Interoceptive Awareness (MAIA)	**NOT** used in this study
5	The Social Economic Status	**USED** in this study
6	Mykinso Pro Questionnaire (e.g., lifestyle, dietary intake, and physical disease)	**USED** in this study

Details and Items of the Questionnaire

1 The Parenting Stress Index (PSI) — NOT used in this study

Reference:

English original:
Abidin, R. R. Parenting Stress Index: Manual, Administration Booklet and Research Update. (Pediatric Psychology Press, 1983).

Japanese version:
Narama, M. et al. Validity and reliability of the Japanese version of the Parenting Stress Index. The Journal of Child Health 58, 610-616 (1999).

This questionnaire requires a purchase for use, and we are not permitted to disclose all of its items publicly.
Below, we provide the URL of the website where we purchased the Japanese version of the PSI questionnaire.
https://www.saccess55.co.jp/kobetu/detail/psi.html

2 The Beck Depression Inventory-II (BDI-II) — USED in this study

Reference:

English original:
Beck, A. T., Ward, C. H., Mendelson, M., Mock, J. & Erbaugh, J. An inventory for measuring depression. Archives of General Psychiatry 4, 561–571 (1961). doi: 10.1001/archpsyc.1961.01710120031004, Pubmed:13688369.

Japanese version:
Kojima, M. & Furukawa, T. Manual for the Beck Depression Inventory-II. (Nihon Bunka Kagakusha, 2003).

This questionnaire requires a purchase for use, and we are not permitted to disclose all of its items publicly.
Below, we provide the URL of the website where we purchased the Japanese version of the BDI-II questionnaire.
https://www.saccess55.co.jp/kobetu/detail/bdi.html

3 The Multidimensional Physical Scale (MDPS) — USED in this study

Reference:

Takeuchi, M., Matsunaga, M., Egashira, R., Miyake, A., Yasuno, F., Nakano, M., Moriguchi, M., Tonari, S., Hotta, S., Hayashi, H., Saito, H., Myowa, M. & Hagihara, K. A multidimensional physical scale is a useful screening test for mild depression associated with childcare in Japanese child-rearing women. Frontiers in Psychiatry 13, 969833 (2022). doi: 10.3389/fpsyt.2022.969833, Pubmed:36532195.

Please circle the one that applies to you from the following options: Yes, Sometimes, or No.

1. I get tired more easily than before.
2. I am sleepy after a meal.
3. I feel sluggish in my hands or legs.
4. I have a sensation of a lump or foreigh body in the throat.
5. I feel that I haven't completely emptied my bladder after urination.
6. I feel bloated.
7. My hands and legs feel cold.
8. My hair loss has become serious.
9. I suffer from dry skin.
10. I suffer from vaginal itchiness or dryness.
11. I have bags under my eyes.
12. I have age spots.
13. I have a rough skin.
14. I have hemorrhoids.
15. I have headaches.
16. I feel dizzy.
17. I have swelling.

4 Multidimensional Assessment of Interoceptive Awareness (MAIA) NOT used in this study

Reference:

Mehling, W.E., Price, C., Daubenmier, J.J., Acree, M., Bartmess, E., Stewart, A. The Multidimensional Assessment of Interoceptive Awareness (MAIA). PLoS ONE 7(11): e48230 (2012) . doi:10.1371/journal.pone.0048230

http://www.osher.ucsf.edu/maia/

Below you will find a list of statements. Please indicate how often each statement applies to you generally in daily life.

1. When I am tense I notice where the tension is located in my body.
2. I notice when I am uncomfortable in my body.
3. I notice where in my body I am comfortable.
4. I notice changes in my breathing, such as whether it slows down or speeds up.
5. I do not notice (I ignore) physical tension or discomfort until they become more severe.
6. I distract myself from sensations of discomfort.
7. When I feel pain or discomfort, I try to power through it.
8. When I feel physical pain, I become upset.
9. I start to worry that something is wrong if I feel any discomfort.
10. I can notice an unpleasant body sensation without worrying about it.
11. I can pay attention to my breath without being distracted by things happening around me.
12. I can maintain awareness of my inner bodily sensations even when there is a lot going on around me.
13. When I am in conversation with someone, I can pay attention to my posture.
14. I can return awareness to my body if I am distracted.
15. I can refocus my attention from thinking to sensing my body.
16. I can maintain awareness of my whole body even when a part of me is in pain or discomfort.
17. I am able to consciously focus on my body as a whole.
18. I notice how my body changes when I am angry.
19. When something is wrong in my life I can feel it in my body.
20. I notice that my body feels different after a peaceful experience.
21. I notice that my breathing becomes free and easy when I feel comfortable.
22. I notice how my body changes when I feel happy / joyful.
23. When I feel overwhelmed I can find a calm place inside.
24. When I bring awareness to my body I feel a sense of calm.
25. I can use my breath to reduce tension.
26. When I am caught up in thoughts, I can calm my mind by focusing on my body/breathing.
27. I listen for information from my body about my emotional state.
28. When I am upset, I take time to explore how my body feels.
29. I listen to my body to inform me about what to do.
30. I am at home in my body.
31. I feel my body is a safe place.
32. I trust my body sensations.

5 The Social Economic Status USED in this study

1. Please answer about your educational background
1-1. What is your highest level of completed education?
(Please circle only one)
Junior High School
High School
Vocational School
Junior College
Four-year University
Graduate School (Master's / Doctoral program)

1-2. How many total years of formal education have you received?
Answer: ______years

Example: Elementary (6), Junior High (3), High School (3), Vocational School (2) = "14"

1-2-2. Are you currently a student (including graduate students)?
Yes / No

If you answered "Yes," please answer the following:

How many days per week do you engage in academic or research activities?
Answer: ______days/week

On average, how many hours per day do you study or conduct research?
Answer: Approx. ______ hours/day

2. Please answer about your current work status (including part-time jobs)
(Please respond based on your average work situation over the past month)

2-1. Are you currently working (including working outside the home)?
Yes / No / On maternity or childcare leave

Note: If you are working from home due to COVID-19, or self-employed and working from home, please choose "Yes."

2-2. If comfortable, please write your job title or occupation:
Answer: ____________________________

2-3. If you are working, how many days per week do you work?
Answer: ______days/week

2-4. If you are working, what is your average working time per day?
Answer: Approx. ______hours/day

3. Annual Personal Income (including all sources)
(Please circle one of the following ranges)
Under 1,000,000 yen
1,000,000 – 2,000,000 yen
2,000,000 – 3,000,000 yen
3,000,000 – 4,000,000 yen
4,000,000 – 5,000,000 yen
5,000,000 – 6,000,000 yen
6,000,000 – 7,000,000 yen
7,000,000 – 8,000,000 yen
8,000,000 – 9,000,000 yen
9,000,000 – 10,000,000 yen
10,000,000 – 15,000,000 yen
Over 15,000,000 yen

4. Household Composition and Income

Number of household members (excluding yourself): ______ people

Relationship to household head (e.g., spouse, parent): ______________________

6 Mykinso Pro Questionnaire NOT used in this study

This was developed by the company (Cykinso, Tokyo, Japan) that analyzes microbiota.
We disclose the questions, but protect the choice of answers.

Q1 Skin type / skin concerns (Multiple answers allowed)
Q2 Do you have any allergies? (Multiple answers allowed)
Q3 Smoking habits / history
Q4 Drinking habits / history
Q5 Have you been doing regular exercise / sports for the past 3 months or more?
Q5 Have you been doing regular exercise / sports for the past 3 months or more?
Q6 Average daily steps
Q7 Sleep duration
Q8 Sleep quality
Q9 What has been your average bowel movement frequency in the last 3 months?
Q10 Regarding your stool condition in the last 3 months, please select the most frequent one.
Q11 Have you been diagnosed with bacterial enteritis in the last 3 months?
Q12 Have you taken any antimicrobial drugs (antibiotics) within 3 months before the stool collection?
Q13 Do you currently have any concerns about your stomach? (Multiple answers allowed)
Q14 Eating frequency (Multiple answers allowed)
Q15 Frequency of eating out for regular meals (breakfast, lunch, dinner)
Q16 Frequency of eating ready-made meals for regular meals (breakfast, lunch, dinner)
Q17 How often did you eat the following foods during the week before collecting the stool sample?

Food items/groups for Q17
Polished rice, bread, noodles, rice cake
Whole grains including brown rice, mixed grains and bran
Root vegetables (such as burdock root, carrots, daikon radish)
Green and yellow vegetables (such as bell peppers, pumpkins, spinach)
Non-green-and-yellow vegetables (such as lettuce, cabbage, cucumber)
Fruits
Meat (including ham and sausage)
Seafood (including salmon roe and cod roe)
Eggs (such as chicken eggs, quail eggs)
Milk and cheese
Yogurt and probiotic drinks
Tofu, soy milk, okara (soy pulp), kinako (roasted soybean flour)
Natto
Japanese pickles
Seaweed (wakame, kombu, hijiki, mozuku, mekabu, agar)
Edible mushrooms
Snack
Sweetened beverages (such as juice, sugary carbonated drinks, and coffee with sugar)

Q18 Past medical history / procedures, etc. Please check all that apply to your current and past health conditions, and provide details in parentheses where possible.

Q19 Are you currently taking any medications (including prescription and over-the-counter drugs)? (Multiple answers allowed)

Q20 Have you been regularly taking any supplements for the past month or more (4 or more days a week)? (Multiple answers allowed)

Supplementary Table 8. Genera Prevalent in the Microbiomes in This Study

Prevalent microbiota_genus	Kingdom	Phylum	Class	Order	Family	Genus
Actinomyces	Bacteria	Actinobacteria	Actinobacteria	Actinomycetales	Actinomycetaceae	Actinomyces
Agathobacter	Bacteria	Firmicutes	Clostridia	Clostridiales	Lachnospiraceae	Agathobacter
Alistipes	Bacteria	Bacteroidetes	Bacteroidia	Bacteroidales	Rikenellaceae	Alistipes
Anaerostipes	Bacteria	Firmicutes	Clostridia	Clostridiales	Lachnospiraceae	Anaerostipes
Bacteroides	Bacteria	Bacteroidetes	Bacteroidia	Bacteroidales	Bacteroidaceae	Bacteroides
Bifidobacterium	Bacteria	Actinobacteria	Actinobacteria	Bifidobacteriales	Bifidobacteriaceae	Bifidobacterium
Bilophila	Bacteria	Proteobacteria	Deltaproteobacteria	Desulfovibrionales	Desulfovibrionaceae	Bilophila
Blautia	Bacteria	Firmicutes	Clostridia	Clostridiales	Lachnospiraceae	Blautia
Butyricicoccus	Bacteria	Firmicutes	Clostridia	Clostridiales	Ruminococcaceae	Butyricicoccus
Colidextribacter	Bacteria	Firmicutes	Clostridia	Clostridiales	Oscillospiraceae	Colidextribacter
Collinsella	Bacteria	Actinobacteria	Coriobacteriia	Coriobacteriales	Coriobacteriaceae	Collinsella
Dorea	Bacteria	Firmicutes	Clostridia	Clostridiales	Lachnospiraceae	Dorea
Eggerthella	Bacteria	Actinobacteria	Coriobacteriia	Coriobacteriales	Coriobacteriaceae	Eggerthella
Erysipelatoclostridium	Bacteria	Firmicutes	Bacilli	Erysipelotrichales	Erysipelatoclostridiaceae	Erysipelatoclostridium
Escherichia-Shigella	Bacteria	Proteobacteria	Gammaproteobacteria	Enterobacteriales	Enterobacteriaceae	Escherichia-Shigella
Faecalibacterium	Bacteria	Firmicutes	Clostridia	Clostridiales	Ruminococcaceae	Faecalibacterium
Flavonifractor	Bacteria	Firmicutes	Clostridia	Clostridiales	Oscillospiraceae	Flavonifractor
Fusicatenibacter	Bacteria	Firmicutes	Clostridia	Clostridiales	Lachnospiraceae	Fusicatenibacter
Intestinibacter	Bacteria	Firmicutes	Clostridia	Clostridiales	Clostridiaceae	Intestinibacter
Lachnoclostridium	Bacteria	Firmicutes	Clostridia	Clostridiales	Lachnospiraceae	Lachnoclostridium
Lachnospira	Bacteria	Firmicutes	Clostridia	Clostridiales	Lachnospiraceae	Lachnospira
Lachnospiraceae_UCG-004	Bacteria	Firmicutes	Clostridia	Clostridiales	Lachnospiraceae	UCG-004
Monoglobus	Bacteria	Firmicutes	Clostridia	Monoglobales	Monoglobaceae	Monoglobus
Negativibacillus	Bacteria	Firmicutes	Clostridia	Clostridiales	Ruminococcaceae	Negativibacillus
Odoribacter	Bacteria	Bacteroidetes	Bacteroidia	Bacteroidales	Marinifilaceae	Odoribacter
Oscillibacter	Bacteria	Firmicutes	Clostridia	Clostridiales	Oscillospiraceae	Oscillibacter
Parabacteroides	Bacteria	Bacteroidetes	Bacteroidia	Bacteroidales	Tannerellaceae	Parabacteroides
Parasutterella	Bacteria	Proteobacteria	Gammaproteobacteria	Burkholderiales	Sutterellaceae	Parasutterella
Phascolarctobacterium	Bacteria	Firmicutes	Negativicutes	Acidaminococcales	Acidaminococcaceae	Phascolarctobacterium
Romboutsia	Bacteria	Firmicutes	Clostridia	Clostridiales	Clostridiaceae	Romboutsia
Roseburia	Bacteria	Firmicutes	Clostridia	Clostridiales	Lachnospiraceae	Roseburia
Sellimonas	Bacteria	Firmicutes	Clostridia	Clostridiales	Lachnospiraceae	Sellimonas
Streptococcus	Bacteria	Firmicutes	Bacilli	Lactobacillales	Streptococcaceae	Streptococcus
Subdoligranulum	Bacteria	Firmicutes	Clostridia	Clostridiales	Ruminococcaceae	Subdoligranulum
Sutterella	Bacteria	Proteobacteria	Gammaproteobacteria	Burkholderiales	Sutterellaceae	Sutterella
Turicibacter	Bacteria	Firmicutes	Bacilli	Erysipelotrichales	Erysipelotrichaceae	Turicibacter
Tuzzerella	Bacteria	Firmicutes	Clostridia	Clostridiales	Lachnospiraceae	Tuzzerella
UBA1819	Bacteria	Firmicutes	Clostridia	Clostridiales	Ruminococcaceae	UBA1819
Veillonella	Bacteria	Firmicutes	Negativicutes	Veillonellales	Veillonellaceae	Veillonella
